# Global estimates of rehabilitation needs and disease burden in tracheal, bronchus, and lung cancer from 1990 to 2019 and projections to 2045 based on the global burden of disease study 2019

**DOI:** 10.3389/fonc.2023.1152209

**Published:** 2023-06-29

**Authors:** Xigui Lai, Conghui Li, Yao Yang, Mingyuan Niu, Yujie Yang, Shanshan Gu, Weiqian Hou, Lili Chen, Yi Zhu

**Affiliations:** ^1^ Department of Musculoskeletal Pain Rehabilitation, The Fifth Affiliated Hospital of Zhengzhou University, Zhengzhou, Henan, China; ^2^ School of Exercise and Health, Shanghai University of Sport, Shanghai, China; ^3^ Academy of Medical Sciences, Zhengzhou University, Zhengzhou, Henan, China; ^4^ Department of Computer Science, University of Waikato, Hamilton, New Zealand; ^5^ School of Rehabilitation Sciences and Engineering, University of Health and Rehabilitation Sciences, Qingdao, Shandong, China; ^6^ Department of Physical Therapy, University of Toronto, Toronto, ON, Canada; ^7^ Department of Rehabilitation Medicine, Hainan Cancer Hospital, Haikou, Hainan, China

**Keywords:** years lived with disability, prevalence, rehabilitation need, projection, TBL cancer

## Abstract

**Background:**

The global cancer burden is substantial and spiraling. Although rehabilitation specialists could offer assistance, oncologic rehabilitation is still underutilized and not a routine part of clinical oncology guidelines worldwide. Global investigations of disease prevalence and years lived with disability (YLDs) for tracheal, bronchus, and lung (TBL) cancer are valuable for facilitating clinical practice improvement and health resource management. The objective of this study is to report the global estimates of rehabilitation needs and disease burden of TBL cancers from 1990 to 2019 and provide predictions for 2045.

**Methods:**

To estimate the need for rehabilitation, the data used from the Global Burden of Disease Study 2019 to calculate the prevalence, YLDs, and the attributable risk factors of TBL cancer. The Bayesian age-period-cohort model and Auto-Regressive Integrated Moving Average model were established to forecast the future health burden. All analyses were done at the global level and then some in the aggregation with the seven World Bank regions. All the data were analyzed by R software (x64 version 4.2.1) and Microsoft Excel (version 2019).

**Results:**

Globally in 2019, 3,212,307 cases of TBL cancer (95% UI 2,937,037-3,488,346) could have benefitted from rehabilitation, contributing to 544,215 (95% UI 396,134-700,099) YLDs. Over the past 30 years, the age-standardized rate (ASR) of prevalence (EAPC = 0.51) and YLDs (EAPC = 0.03) increased. Throughout this period, the global prevalence and YLDs counts were greater in males than females. The ASR of prevalence and YLDs are projected to show a slight downward trend by 2045 on the global scale, the overall prevalence and YLDs due to TBL cancer are likely to increase further, but all indicators show a growing trend in females.

**Conclusion:**

TBL cancer remains one of the major public health issues globally. According to the forecasted results, the burden of YLDs due to TBL cancer will continue to rise, and the increment is higher in females than males. A rising number of patients worldwide will benefit from rehabilitation services in the future to achieve precise control and management throughout the TBL cancer patient lifecycle.

## 1 Introduction

Tracheal, bronchial, and lung (TBL) cancer has the highest mortality rate globally and is the second largest group of new cancer cases (1). In 2019, it was reported that the percentage of disability-adjusted life-years (DALYs) among all diseases for TBL cancer was ranked 17th in all ages and was ranked 5th in 50-74 years (2). It imposes a huge global health threat. In recent years, advances in cancer treatment options and technology have increased life expectancy and reduced the mortality rate of lung cancer. The improved prognosis for lung cancer patients draws the time course of the disease closer to that of chronic diseases (3). However, the malignant disease itself, the burden of cancer-related symptoms, and the side effects of cancer treatment can all impair the quality of life and functional status of lung cancer patients and survivors, even leading to permanent disability (4, 5). According to recent studies, tracheal, bronchial, and lung cancers will cost the global economy $3.9 trillion between 2020 and 2050, placing a heavy financial burden on patients, families, and society (6).

The current global health challenges include the dramatic increase in the incidence of non-communicable chronic diseases and the increasing number of people lived in with restricted function (2, 7), which are particularly pronounced among people with TBL cancer. As disability accounts for a large component of the disease burden, it also accounts for a huge share of health expenditure. Conditions with higher disability weight contributed more to years lived with disability (YLDs) than others, corresponding to more rehabilitation needs. The disability (8) weights for the diagnosis and primary therapy phase, the metastatic phase, and the terminal phase of TBL cancer are 0.28, 0.451, and 0.54, which are greater than the disability weight for severe lower back pain. As a result, there is a growing interest in health policies and interventions that can extend TBL cancer patient’s life and promote health. Strengthening the capacity of health systems to provide rehabilitation services (9, 10) could help reduce the impact of disability and optimize remaining functions. Growing evidence shows that pulmonary rehabilitation is beneficial to patients with lung cancer and cost-effective (11–14). Therefore, pulmonary rehabilitation is gradually incorporated into the multidisciplinary management of lung cancer (15).

Despite the known benefits of rehabilitation for TBL cancer patients which translate to substantial economic benefits across the world, there remains a high prevalence of unmet rehabilitation needs in cancer patients. Cancer patients and survivors face many adverse symptoms related to the disease itself or its treatments. Although there are many types of symptoms, many are physical impairments, such as cachexia, lymphoedema, breathlessness, and limited range of motion in joints (16). The overall improved survival rate in the face of prolonged physical dysfunction creates an immense need for both acute and long-term rehabilitation services (17). However, rehabilitation has not been a priority in cancer care in all countries and remains under-resourced. This is not surprising, given the primary concern of oncologists and patients is to prevent tumor progression or reoccurrence through cancer treatment. There also has been a lack of public awareness about cancer rehabilitation and limited access to cancer rehabilitation specialists (18, 19).

The Global Burden of Disease (GBD) Study 2019 provides annual estimates of health losses from 369 diseases and injuries and associated risk factors for 204 countries and territories from 1990 to 2019 (2). All data are available and accessible to researchers through the GBD online system, facilitating the use of these data to analyze changing trends in different diseases. The traditional measures of disease burden mainly include incidence, prevalence, mortality, etc., which only consider the survival quantity, not the quality of life. Hence, in this study, we use data from the Global Burden of Disease Study 2019 to assess the need for rehabilitation by presenting prevalence and YLDs for TBL cancers (20), thus challenging the current dilemma of cancer rehabilitation.

The purpose of this study is to provide up-to-date insights into the rehabilitation needs and the burden of TBL cancer from 1990 to 2019 and attributable risk factors by gender and age. We also attempt to make projections to 2045, which will help guide the allocation of rehabilitation resources and the formation of a multidisciplinary model of cancer care, as well as precise control and full life-cycle management of TBL cancer. Specifically, we try to answer the following questions:① How large are the rehabilitation needs and disease burden of TBL cancer in 2019 (e.g., in nominal numbers, age-standardized rates), and how have those values evolved since 1990? ②What risk factors contribute to the increase of YLDs in TBL cancer? Which of them is modifiable? ③After 2019, what are the trends of rehabilitation needs and disease burden for the next 26 years?

## 2 Methods

### 2.1 Overview

TBL cancer was identified using the International Classification of Diseases(ICD) codes, Tenth Revision and Ninth Revision (ICD-10 and ICD-9, respectively), which was defined as the ICD-10 code C33, C34–C34.92, Z12.2, Z80.1–Z80.2, Z85.1–Z85.20 and the ICD-9 code 162–162.9, 209.21, V10.1–V10.20, V16.1–V16.2, V16.4–V16.40 (2, 21). The GBD study evaluated 369 diseases and injuries and associated 87 risk factors for 204 countries, 21 regions, and 7 super-regions (2). In the GBD project, uncertainty estimation was performed by generating 1000 draws for each estimate. The custom aggregation for this study was done at the draw level, taking 2.5^th^ and 97.5^th^ percentile to generate 95% uncertainty intervals (UI), standardized by GBD standard population and reported per 100,000 populations. In addition to the absolute number and rate per 100,000 persons, we also applied the age-standardized rate (ASR) per 100,000 persons, including the age-standardized prevalence rate (ASPR) and age-standardized YLDs rate (ASYR), given the heterogeneity in the age structure of the population.

### 2.2 Measures

Previous studies estimated the rehabilitation needs through YLDs (20, 22–24), which is the measure in the GBD study that focuses exclusively on non-fatal health losses. The YLDs refer to years lived with any short-term or long-term health loss weighted for severity by the disability weights. Sequelae were each mapped to a health state with an associated disability weight (8), valuing the severity of the sequela. The four common sequelae for TBL cancers in GBD and their corresponding disability weights were considered for cancer (**Supplementary 1 Table S1**) (2). Disability weight is used to calculate years lived with disability (YLD) for these outcomes in a given population, which represented the magnitude of health loss associated with specific health outcomes. The weights are measured on a scale from 0 to 1, where 0 equals a state of full health and 1 equals death. The prevalence is a measure of disease burden, which is defined as the proportion of people in a population who are a case of a disease, injury, or sequela.

### 2.3 Data sources

Previous studies have described the detailed methodology of processes for estimating the burden and rehabilitation needs of cancers, and risk factor quantification in the GBD 2019 Study (2, 20, 25). We extracted data on the global prevalence and YLDs of TBL cancer (1990 to 2019) from the official website of the GBD 2019 Study (2, 26), which is available from the Institute for Health Metrics and Evaluation (IHME) for free.

We chose “Global” and seven “World Bank Regions” (“East Asia & Pacific –WB”, “Europe & Central Asia –WB”, “Latin America & Caribbean –WB”, “Middle East & North Africa –WB”, “North America”, “South Asia –WB”, “Sub-Saharan Africa -WB”) (See detailed regional division for **Supplementary 2**) from the database as the location, “Tracheal, bronchus, and lung cancer” for the cause, “Prevalence” and “YLDs (Years Lived with Disability)” for measures. In this study, we present the prevalence and YLDs for TBL cancer globally by sex and age. The study also presents the percentage change of these indicators from 1990 to 2019 to reflect the trends in cancer burden. And to identify risk factors of YLDs for TBL cancer, we chose “Risk factor” from the database at the GBD Estimate, “percent” for the metric, and “YLDs (Years Lived with Disability)” measures. These risk factors were smoking, ambient particulate matter, high fasting plasma glucose levels, low fruit and vegetable consumption, and exposure to pollutants, such as occupational secondhand smoke, asbestos, radon, household air pollution, occupation silica, occupational nickel, occupational arsenic, occupational diesel, and occupational polycyclic aromatic hydrocarbons, which defined in detail and their relative risk for TBL cancer could be found in a previous article (25).

Additional information including data sources used results, and analytical code can be found at http://ghdx.healthdata.org/gbd-results-tool and https://vizhub.healthdata.org/gbd-compare/. The estimated population of Global was taken from the United Nations World Population Prospects 2019 Revision, by year (up to 2100), age, and sex (https://population.un.org/wpp/Download/Standard/Population/).

### 2.4 Data management and analysis

All data analyses and image presentations are conducted by Microsoft Excel (version 2019) and the open-source software R (version 4.2.1). Packages included ggplot2 and RColorBrewer.

#### 2.4.1 Description of the past and the present

We showed the secular trend in TBL cancer burden along with its attributable risk factors by sex, year, region, and age. Data on the prevalence and YLDs of TBL cancer were analyzed descriptively. Cases were divided into 5-year age groups to describe the number of prevalence and YLDs in 1990 and 2019.

#### 2.4.2 Analysis of long-term trend

To reflect trends in cancer burden, we also calculated the global change in the number of people with TBL cancer by Microsoft Excel and the estimated annual percentage change (EAPC) by software R in age-standardized prevalence and age-standardized YLDs rates from 1990 to 2019. The EAPC was introduced to measure the temporal trends in age-standardized rates (ASRs). Calculations were based on a regression model fitted to the natural logarithm of the rate, i.e. *y* = *α* + *βx* + *ε*, was fitted to the natural logarithm of the rates, where *y* was referred to ln(ASR), and *x* the calendar year (27). EAPC was calculated as 100 × (exp*^[β]^*− 1) and its 95% confidence intervals (*CI*s) were obtained from the linear model (27). If the EAPC and the corresponding 95% CI are positive, the detection rate increases; if the EAPC and the corresponding 95% CI are negative, the detection rate decreases; otherwise, it is stable.

#### 2.4.3 Risk factors for TBL cancer burden and rehabilitation

To describe the trends in each risk-outcome pair, we obtained the attributable TBL cancer YLDs globally and in the World Bank Region by sex, year, and age. Then we reported the percent of YLDs owing to TBL cancer which was attributable to smoking, ambient particulate matter, high fasting plasma glucose levels, low fruit and vegetable consumption, and exposure to pollutants.

#### 2.4.4 The future projections of rehabilitation needs and disease burden

The Bayesian age-period-cohort (BAPC) model turned out to be the most appropriate statistical method of projecting the cancer burden compared with a generalized additive model, Nordpred model, Joinpoint model, smooth spline model, and Poisson regression, especially for short-term projections (28–30). In accordance with the characteristics of the BAPC model, and to ensure the accuracy of the prediction, and considering that the cancer burden will continue to change with the rapid development of medical treatment and the continuous updating of cancer treatment methods, our study predicts the demand for rehabilitation and the disease burden by 2045. We performed BAPC model analyses by sex using the BAPC package in R integrated with the nested Laplace approximation (INLA) to predict the prevalence and YLDs in age-standardized rates (ASRs) due to TBL cancer from 2019 to 2045, taking into account rates of change and demographic changes, which have been well documented and accepted in previous studies (31–35).

Auto-Regressive Integrated Moving Average (ARIMA) model was widely used in epidemiological studies to predict future trends (36–38). The ARIMA model was built-in R to predict the number of people with the prevalence and YLDs due to TBL cancer from 2019 to 2045, refer to this study (38) for specific sources of R codes. Based on the predicted results, the model was box-tested and p > 0.05 indicates that the model has a good fit effect.

Detailed information about the BAPC model and the ARIMA model, also includes the prediction R codes are given respectively in **Supplementary Materials 3**, **4**. (The prediction plots of our study can be recapitulated from the data information and R codes in these **Supplementary Materials**.)

## 3 Results

### 3.1 The temporal trend in TBL cancer burden and rehabilitation needs in global

On a global scale in 2019, 3,212,307 cases of TBL cancer (95% UI 2,937,037-3,488,346) patients had symptoms that would benefit from rehabilitation services at some point during disease, contributing to 544,215(95% UI 396,134-700,099) YLDs. Over the past 30 years, the prevalence and YLDs of TBL cancer had increased by the 1.32-fold and 1.07-fold increase from 1990, respectively, while the ASPR (EAPC = 0.51) and ASYR (EAPC = 0.03) increased slightly from 1990 to 2019. Throughout this time, the global prevalence and YLDs were greater in males than in females. ASPR (EAPC = -0.18) and ASYR (EAPC =-0.33) in males showed modest declines since 1990, while in females there was an upward trend by ASPR (EAPC =0.45) and ASYR (EAPC =0.8). (Table1)

In 2019, among World Bank (WB) regions, the East Asia & Pacific-WB had the highest need for rehabilitation services with the highest absolute number (prevalence=1,658,888; YLDs=279,857) of TBL cancer, followed by the Europe & Central Asia-WB (prevalence=720,942; YLDs=122,125), while the lowest absolute number (prevalence= 46,144; YLDs=9,848) in Middle East & North Africa-WB. Furthermore, the number of TBL cancer patients increased in all WB regions, with the highest increase in East Asia & Pacific-WB (2.63-fold), while the ASPR and ASYR decreased respectively in Latin America & Caribbean-WB (EAPC =-0.5; -0.68) and North America (EAPC = -0.69; -1.09) from 1990 to 2019. The trends of the ASPR (EAPC =0.2) and ASYR (EAPC = -0.36) are opposite in Europe & Central Asia-WB (**Table 1**).

**Table 1:** The number of prevalence and YLDs of tracheal, bronchial and lung cancer in global and regions in 1990 and 2019, the change in counts and the estimated annual percentage changes from 1990 to 2019.

Characteristics	Prevalence				YLDs			
	1990 Numbers (95% UI)	2019 Numbers (95% UI)	1990–2019 Change in Counts	1990–2019 EAPC in ASR (95% CI)	1990 Numbers (95% UI)	2019 Numbers (95% UI)	1990–2019 Change in Counts	1990–2019 EAPC in ASR (95% CI)
**Global**	1385579 (1334784-1443423)	3212307 (2937037-3488346)	1.32	0.51 (-0.98-2.02)	262763 (190730-331420)	544215 (396134-700099)	1.07	0.03 (-3.62-3.83)
Sex								
Female	375419 (358092-392604)	1102899 (989626-1214657)	1.94	0.45 (-1.13-2.05)	69319 (49994-87919)	178776 (128528-231634)	1.58	0.8 (-4.14-5.99)
Male	1010159 (965158-1062112)	2109409 (1895836-2337805)	1.09	-0.19 (-2.5-2.17)	193445 (140046-244602)	365439 (266230-468546)	0.83	-0.33 (-3.29-2.73)
World Bank Regions								
East Asia & Pacific-WB	457428 (414828-500717)	1658888 (1449615-1868682)	2.63	1.84 (1.7-1.98)	91001 (65141-117611)	279857 (201246-364959)	2.08	1.14 (1.02-1.26)
Europe & Central Asia-WB	486659 (476662-495194)	720942 (650006-797695)	0.48	0.2 (0.1-0.29)	93632 (67661-118339)	122125 (87247-157531)	0.30	-0.36 (-0.44–0.29)
Latin America & Caribbean-WB	51560 (50313-52586)	111264 (98714-124508)	1.16	-0.5 (-0.53–0.46)	11241 (8016-14274)	23310 (16444-30696)	1.07	-0.68 (-0.71–0.65)
Middle East & North Africa-WB	15370 (13513-17261)	46144 (40204-52944)	2.00	0.56 (0.5-0.62)	3421 (2417-4448)	9848 (6965-13127)	1.88	0.4 (0.35-0.45)
North America	306727 (298508-313496)	499549 (437947-571551)	0.63	-0.69 (-0.89–0.49)	47990 (35383-59946)	69595 (50234-90028)	0.45	-1.09 (-1.25–0.92)
South Asia-WB	45125 (38920-51747)	125826 (108230-143575)	1.79	0.37 (0.3-0.44)	10283 (7039-13845)	28276 (19633-37629)	1.75	0.25 (0.18-0.31)
Sub-Saharan Africa-WB	21483 (18020-25341)	46870 (40601-54173)	1.18	0.05 (-0.01-0.11)	4946 (3402-6744)	10663 (7463-14275)	1.16	0.04 (-0.02-0.09)

Generated from data available from http://ghdx.healthdata.org/gbd-results-tool.

UI, uncertainty interval; ASR, age standardized rate; EAPC, estimated annual percentage change; CI, confidence interval; YLDs, years lived with disability; WB, World Bank.

**Figure 1:**
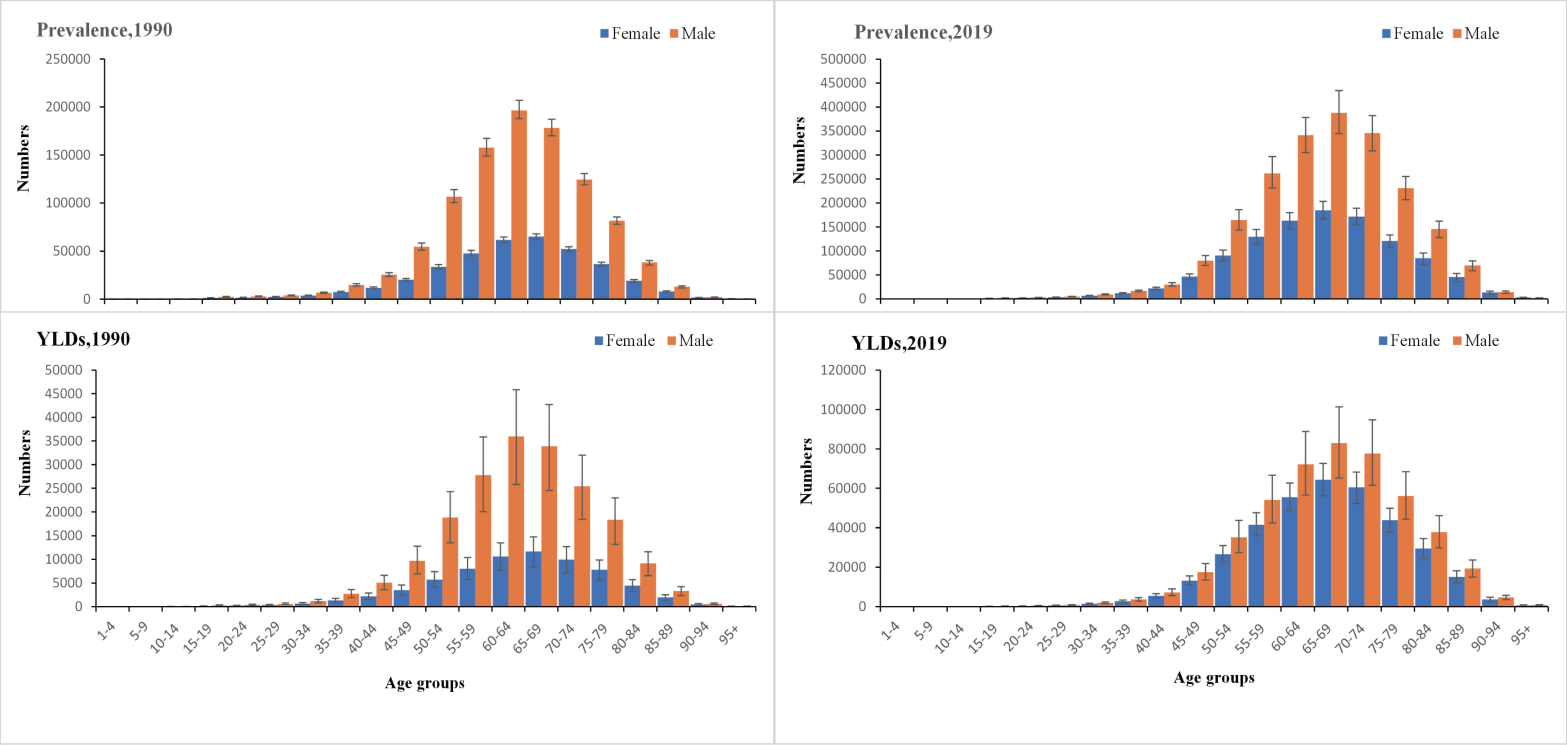
The global number of prevalent cases with conditions that would benefit from rehabilitation and the corresponding years of life lived with disability by age and sex with 95% uncertainty intervals, 1990 and 2019. YLDs, years lived with disability.

Regardless of time and age groups, the males had a higher prevalence and YLDs than females. Globally, both prevalence and YLDs were highest for people aged 45–80 years old and lowest in people aged less than 25 or 95 and older. The female YLDs in the age group 35-90 years old was increasing, gradually narrowing the gap between females and males, especially the significant change between 50-85 years old. In addition, TBL cancer patients between 50-85 years have shown a greater need for rehabilitation (**Figure 1**).

### 3.2 Projections of TBL cancer burden and rehabilitation need *via* prevalence and YLDs from 2020 to 2045

**Figure 2:**
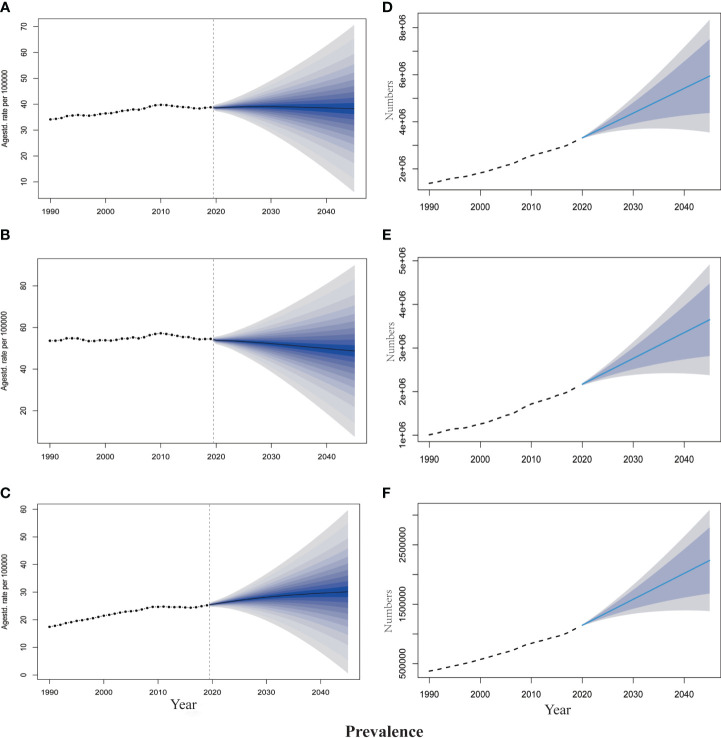
The projection of TBL cancer prevalence from 2020 to 2045 worldwide. **(A)** The age-standardized prevalence rate for all gender. **(B)** The age-standardized prevalence rate for males. **(C)** The age-standardized prevalence rate for females. **(D)** The projected numbers of prevalence for all gender. **(E)** The projected numbers of prevalence for males; **(F)** The projected numbers of prevalence for females. The predictive mean is shown as a solid line. The dotted line represents the observational values from the GBD dataset. The vertical dashed line indicates where the prediction starts. The predictive mean value is shown as a solid black line. Agestd., Age standardized.

**Figure 3:**
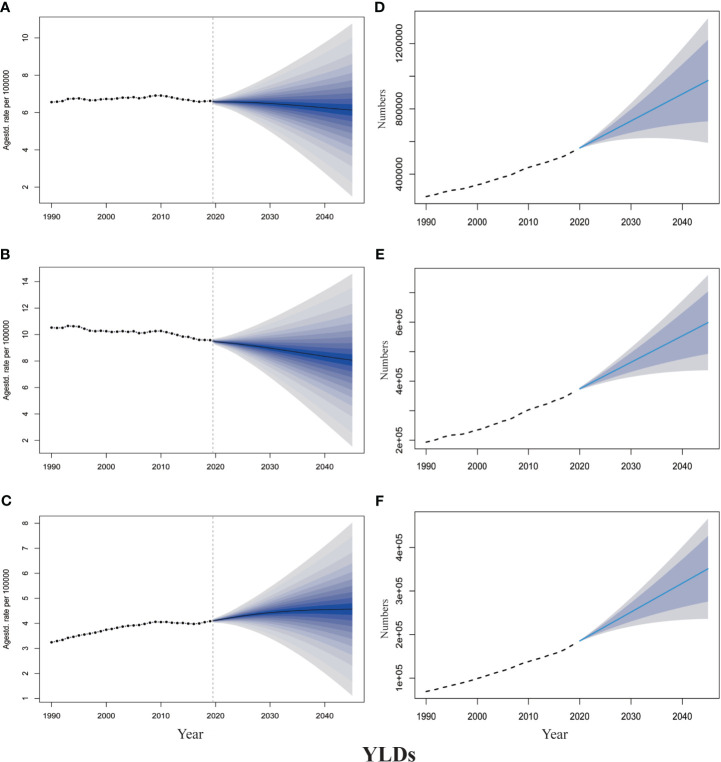
The projection of TBL cancer years lived with disability (YLDs) from 2020 to 2045 worldwide. **(A)** The age-standardized YLDs rate for all gender. **(B)** The age-standardized YLDs rate for males. **(C)** The age-standardized YLDs rate for females. **(D)** The projected numbers of YLDs for all gender. **(E)** The projected numbers of YLDs for males; **(F)** The projected numbers of YLDs for females. The predictive mean is shown as a solid line. The dotted line represents the observational values from the GBD dataset. The vertical dashed line indicates where the prediction starts. The predictive mean value is shown as a solid black line. Agestd., Age standardized.

The projections of TBL cancer prevalence, YLDs, and corresponding ASRs for 2020–2045 to reflect changes in cancer burden and rehabilitation needs were presented in **Figures 2**, **3**. From 2020 to 2045, ASPR and ASYR of both genders and male TBL cancer patients would decrease, and males showed a more noticeable decline than all gender (**Figures 2A, B**, **3A, B**). However, as for female TBL cancer, there would be a slight upward trend in ASPR and ASYR (**Figures 2C**, **3C**). The projected number of TBL cancer prevalent cases and YLDs would increase steadily (**Figures 2D–F**, **3D–F**), and the absolute number would be much larger in males than that in females, but women had a higher increment than men (**Supplementary Material 5**). By the year 2045, there would be 3,647,906 male TBL cancer cases and 598,340 YLDs in males, 2,237,459 female prevalent cases, and 350,986 YLDs in females (**Figures 3E, F**).

### 3.3 Proportion of YLDs Attributable to risk factors in 2019

**Figure 4:**
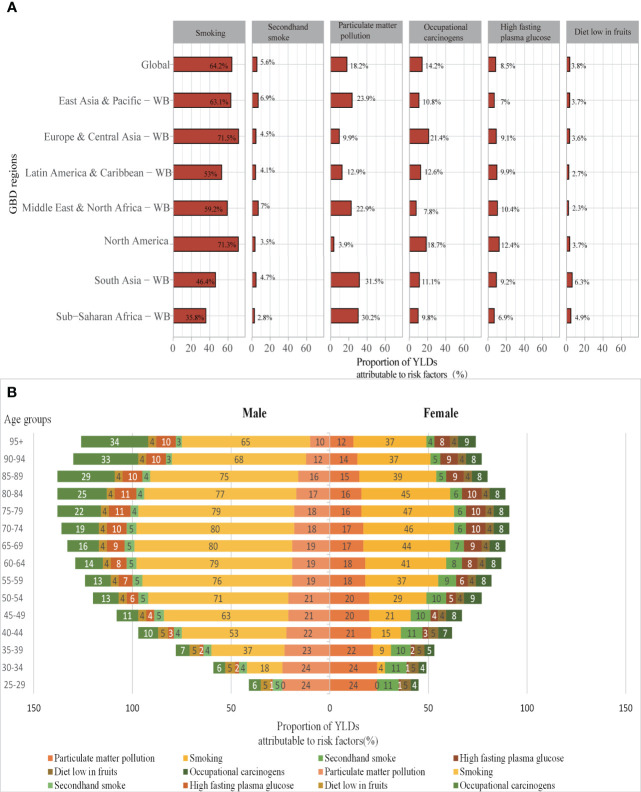
**(A)** Proportions of TBL cancer YLDs attributable to risk factors for global and World Bank regions in 2019; **(B)** Proportions of YLDs attributable to risk factors by age and sex in 2019. YLDs, years lived with disability; WB, world bank; GBD, Global Burden of Diseases.

At the global level, a substantial proportion of YLDs was attributable to the six risk factors for which GBD estimates were available, including 64.2% (95% UI 62.1–66.4) attributable to smoking, 18.2% (13.9–22.7) to particulate matter pollution, 14.2% (11.0–17.5) to occupational carcinogens, 8.5% (2.0–18.3) to high fasting plasma glucose, 5.6% (3.4–8.3) to second-hand smoke, and 3.8% (1.1–5.6) to diet low in fruits (**Figure 4A**). Smoking was the highest risk factor in all regions and the impact of the rest of these risk factors varied by region. For example, the impact of occupational carcinogens was highest in Europe & Central Asia-WB (21.4% of YLDs were attributable to occupational carcinogens) and North America (18.7%), where occupational carcinogens are still prevalent, and lowest in the Middle East & North Africa-WB (7.8%). Likewise, the impact of particulate matter pollution was the highest in South Asia-WB (31.5% of YLDs attributable to particulate matter pollution) and Sub-Saharan Africa-WB (30.2%), and lowest in North America (3.9%). The proportion of YLDs attributable to a diet low in fruit was generally low (only 4.9%).

Due to differences in lifestyles and occupational exposure, the risk factors to which the genders and the various age groups were exposed varied substantially. Smoking is the most significant risk factor for TBL cancer disability in both sexes, followed by particulate matter pollution. In addition, the common risk factors for males were occupational carcinogens and high fasting plasma glucose, while females were second-hand smoke and high fasting plasma glucose. Smoking is a significant risk factor for men over 40 years of age, the proportion of YLDs attributable to smoking is over 50% and up to 79%. While in women it is mainly concentrated in 60-85 years of age, with the highest at 47%. The main risk factors for young and middle-aged women aged 25-50 are high fasting glucose and second-hand smoke. The proportion of risk factors for occupational carcinogens in males is increasing with age due to long-term exposure to carcinogens in the work environment and the development of TBL cancer after a long latency period (**Figure 4B**).

## 4 Discussion

### 4.1 Summary of main findings

The increasing disease burden of TBL cancer is a major concern for healthcare institutions worldwide. Based on the GBD 2019 data, this study provides a multifaceted analysis of the current global TBL cancer burden and rehabilitation needs. Prevalence and YLDs are the two indicators used to analyze the long-term trends of cancer burden and rehabilitation needs from 2020-2045. We estimated the need for rehabilitation services for TBL cancer globally, and our findings suggest that 3,212,307 cases of TBL cancer (95% UI 2,937,037-3,488,346) could benefit from rehabilitation services. According to the sequelae of TBL cancer and corresponding disability weights in the GBD 2019 study, this finding shows that the majority of TBL patients worldwide could benefit from rehabilitation at some point during their disease, and this result contradicts the commonly held view that rehabilitation is a service for only a few.

Our results found that the number of prevalence and YLDs due to TBL cancer increased more than 1-fold globally from 1990 to 2019. According to the age-sex pattern of prevalence and YLDs, more male patients developed lung cancer than female patients. The global ASPR and ASYR did not change significantly overall, but an increasing trend was observed in women, not men. Although the projected ASPR and ASYR show a slight downward trend by 2045 globally, the total number of prevalence and YLDs due to TBL cancer is likely to increase further, which predicted the trend would be consistent with the occupational carcinogenic lung cancer burden (mortality and disability-adjusted life years) in China (34). Predicted results from another study similarly confirm that the burden of lung cancer in China has been increasing (35). However, prevention and control of TBL cancers are complicated by significant differences in the global burden of TBL cancers due to differences in specific pathological patterns, risk factors, regions, sex, and age groups. These temporal trends suggest that TBL cancers remain a major disease burden worldwide, and their total cancer burden and rehabilitation needs are likely to continue to increase. This further indicates that without the implementation of effective rehabilitation interventions for TBL cancers globally in the future, the burden of YLDs due to TBL cancers will further increase in the global population, especially in women, along with population aging.

### 4.2 The burden of TBL cancer in the female population cannot be ignored

According to the predicted results of this study, the number of prevalence and YLDs, as well as ASPR and ASYR of TBL cancer in females from 1990 to 2045 was rising. In contrast, the ASPR and ASYR show a downward trend for males. Attention should be paid to the burden of TBL cancer in females. The rising rates of lung cancer in women have also been attributed to genetic variants, hormonal factors, environmental exposures, and oncogenic viruses (39, 40). Genetic and biological differences between males and females could explain the differences in lung cancer incidence and mortality, but many questions remain unanswered, suggesting that women should be screened at lower pack years than men and younger ages (41). Efforts to prevent smoking initiation for adolescent girls and to encourage adult smokers to quit are most likely to reduce the burden of lung cancer in women (42).

### 4.3 The attributable YLDs risk factor: high fasting plasma glucose

This study identified modifiable risk factors intending to be able to reduce YLDs in TBL cancer through effective intervention strategies in clinical settings. As far as we know, smoking (64.2%), particulate matter pollution (18.2%), and occupational carcinogens (14.2%) are the top three attributions to YLDs of global TBL cancer for both sexes in 2019. This result is different from the proportion of the top three risk factors for DALYs: smoking (62.4%), ambient particulate matter (15.3%), and high fasting plasma glucose (9.9%) (43). Smoking is the most recognized risk factor for developing TBL cancer, increasing the burden of the disease (44). According to the 2019 global, regional, and national cancer burden study, almost 1 in 4 deaths and 1 in 5 DALYs were found to be due to smoking, smoking remains a significant risk factor (45). For TBL cancer prevention, the immediate priority is still to control smoking rates and minimize exposure to second-hand smoke (21). Besides smoking, high fasting plasma glucose is another risk factor worthy of attention. Furthermore, in a recent study, high fasting plasma glucose was also an essential risk factor affecting disability-adjusted life years attributable to cancer (46). The results of a study found in 2019 that high fasting plasma was associated with a greater burden of cancer, especially in older men living in developed countries (47). Hyperglycaemia may be one of the direct biological mechanisms underlying the association between diabetes and cancer. Hyperglycaemia is associated with the level of extracellular glucose to the dynamic regulation of 5hmC through the glucose–AMPK–TET2–5hmC axis (48). The high glucose levels can provide a nutritional base to maximize tumor cell proliferation (49). Patients with diabetes mellitus should accept the recommended age and gender-appropriate cancer screening to promote primary prevention and early detection. In addition, cancer should be screened for in routine diabetes assessments (50).

### 4.4 Early interventions in cancer rehabilitation for the management throughout TBL cancer life-cycle

The need for rehabilitation has been growing globally as the prevalence of disability has risen. A study (23) found that the number of YLDs worldwide has increased by 66% since 1990, and the world’s per capita physical need for rehabilitation has increased by 17%. Usually, people believe only antineoplastic treatment is valued for TBL cancer patients. And rehabilitation services are unnecessary, disadvantageous, and burdensome (51). But our findings challenged this view, as we show that 3,212,307 cases may need rehabilitation in the study. Furthermore, there is growing evidence that many TBL cancer patients will suffer from sequelae of varying severity afterward, increasing the global demand for rehabilitation services. More research and development investment is needed to identify new, more effective intervention strategies. As the prevalence of TBL cancer continues to rise and the population ages, the number of cancer patients who will benefit from rehabilitation close to home will also increase.

Many oncology guidelines include recommendations for rehabilitation referrals and interventions, demonstrating that rehabilitation is a recognized and necessary service in oncology care. Evidence-based guidelines support the use of rehabilitation assessments and interventions to treat individual physical and cognitive impairments in many different cancer types. They also suggested using the guideline recommendations to manage oncology treatment-related symptoms and conditions in the clinical setting. Encouraging participation in rehabilitation care could optimize function and quality of life for cancer patients and survivors (52). Physiotherapists could play a critical role in lung cancer and the management of lung cancer needs to focus on physiotherapy interventions to improve its cancer treatment-related side effects (53). Palliative care aims to break down cancer treatment barriers, enabling patients to cope with cancer in the later stages of the cancer journey. Integrating function-directed treatments and pulmonary rehabilitation into palliative care may serve as one of those options (54). Therapeutic exercises should be an essential intervention for cancer rehabilitation. There is growing evidence for exercise interventions to reduce cancer morbidity in lung cancer. Exercise could prevent deterioration and maximize or restore physical status before, during, and following treatment (53). Rehabilitation services can be provided before (55–57)and after (58, 59) TBL cancer surgery, during cancer treatment in hospital (60, 61), outpatient (62), community, and at home (63, 64)throughout the full life-cycle of cancer management. The current telehealth trend shows that telecare may be a potential choice for lung cancer during the COVID-19 pandemic (65).

### 4.5 Limitations

Our study presents some limitations. First, it is important to first consider all the limitations presented in the GBD study, which had been emphasized before (2). Indeed, the quality and quantity of estimates available in GBD studies are critical to the accuracy of our estimates. For example, it is not possible to assess the disease burden in countries and regions without an established and organized structure for registering, recording, and reporting diseases. Detection bias may be partly responsible for the observed variation in prevalence and YLDs due to changes in screening programs over time and between countries. In addition, we selected anyone who could benefit from rehabilitation at any time during the disease, which does not imply that all populations with TBL cancer have an urgent need for rehabilitation. Not including all of the potential risk factors for TBL cancer can be another limitation of the GBD study and the current one. Future studies should address these limitations to improve our understanding of the overall disease burden.

## 5 Conclusion

This study provides a global estimate of the need for rehabilitation services for TBL cancer. Our findings suggest that 3,212,307 cases of TBL cancer could benefit from rehabilitation services, which challenge the common view that only a minority of people need rehabilitation. The corresponding increase in ASPR and ASYR were shown for lung cancer occurred from 1990 to 2019 for all genders globally, but projections from 2020 to 2045 showed a decreasing trend in both ASPR and ASYR. In addition, male patients are significantly more affected and have been on the decline, while females have been on the increasing trend. From 1990 to 2045, the number of patients with both sex and number of YLDs has been an increasing trend, which was greater in males than females. The sex-specific differences in risk factors attributed to YLDs underscore the need for targeted strategies to reduce the burden of lung cancer. Our results could help conduct preventive measures for precise control of symptoms and full life-cycle management of TBL cancer. Furthermore, these data could also contribute to the development of health policies, the allocation of rehabilitation resources, and the establishment of multidisciplinary cancer care models.

## Data availability statement

The original contributions presented in the study are included in the article/**Supplementary Material**. Further inquiries can be directed to the corresponding author.

## Ethics statement

Ethical review and approval was not required for the study on human participants in accordance with the local legislation and institutional requirements. Written informed consent from the participants’ legal guardian/next of kin was not required to participate in this study in accordance with the national legislation and the institutional requirements.

## Author contributions

Study concept, design, and supervision: XL and YZ. Refining and modifying R Code: CL, MN, XL, and YaY. Data processing, analysis, and charting: XL. YuY, SG, WH, and LC revised the manuscript for important intellectual content. Drafting of the manuscript: XL. Revision of the manuscript: YZ and XL. All authors have edited, reviewed, and approved the final version of the manuscript.
