# Supplementary material for: Global estimates of rehabilitation needs and disease burden in tracheal, bronchus, and lung cancer from 1990 to 2019 and projections to 2045 based on the global burden of disease study 2019
Source: Front Oncol. 2023 Jun 29;13:1152209. doi: 10.3389/fonc.2023.1152209 (PMC10344363; doi:10.3389/fonc.2023.1152209)
Supplement: Supplementary file 1 [file DataSheet_1.zip › Supplementary Material/Supplementary Material 1.pdf]

Table S1: Sequelae for tracheal, bronchus, and lung cancer and corresponding disability weights in the GBD 2019 Study			
**Sequelae**	**Health state name**	**Lay description**	**DW (95% CI)**
Diagnosis and primary therapy phase	Cancer, diagnosis and primary therapy	Has pain, nausea, fatigue, weight loss and high anxiety.	0.288 (0.193-0.399)
Controlled phase	Generic uncomplicated disease: worry and daily medication	has a chronic disease that requires medication every day and causes some worry but minimal interference with daily activities.	0.049 (0.031-0.072)
Metastatic phase	Cancer, metastatic	has severe pain, extreme fatigue, weight loss and high anxiety.	0.451 (0.307-0.6)
Terminal phase	Terminal phase, with medication (for cancers, end-stage kidney/liver disease)	has lost a lot of weight and regularly uses strong medication to avoid constant pain. The person has no appetite, feels nauseous, and needs to spend most of the day in bed.	0.54 (0.377-0.687)

GBD: Global Burden of Disease

DW: Disability weight

Data from Global Burden of Disease Collaborative Network. Global Burden of Disease Study 2019 (GBD 2019) Disability Weights. Seattle, United States of America: Institute for Health Metrics and Evaluation (IHME), 2020.

https://ghdx.healthdata.org/record/ihme-data/gbd-2019-disability-weights
