# Supplementary material for: Global estimates of rehabilitation needs and disease burden in tracheal, bronchus, and lung cancer from 1990 to 2019 and projections to 2045 based on the global burden of disease study 2019
Source: Front Oncol. 2023 Jun 29;13:1152209. doi: 10.3389/fonc.2023.1152209 (PMC10344363; doi:10.3389/fonc.2023.1152209)
Supplement: Supplementary file 1 [file DataSheet_1.zip › Supplementary Material/Supplementary Material 2.pdf]

The world by region

Classified according to World Bank analytical grouping

- East Asia and Pacific
- Europe and Central Asia
- Latin America and Caribbean
- Middle East and North Africa
- North America
- South Asia
- Sub-Saharan Africa

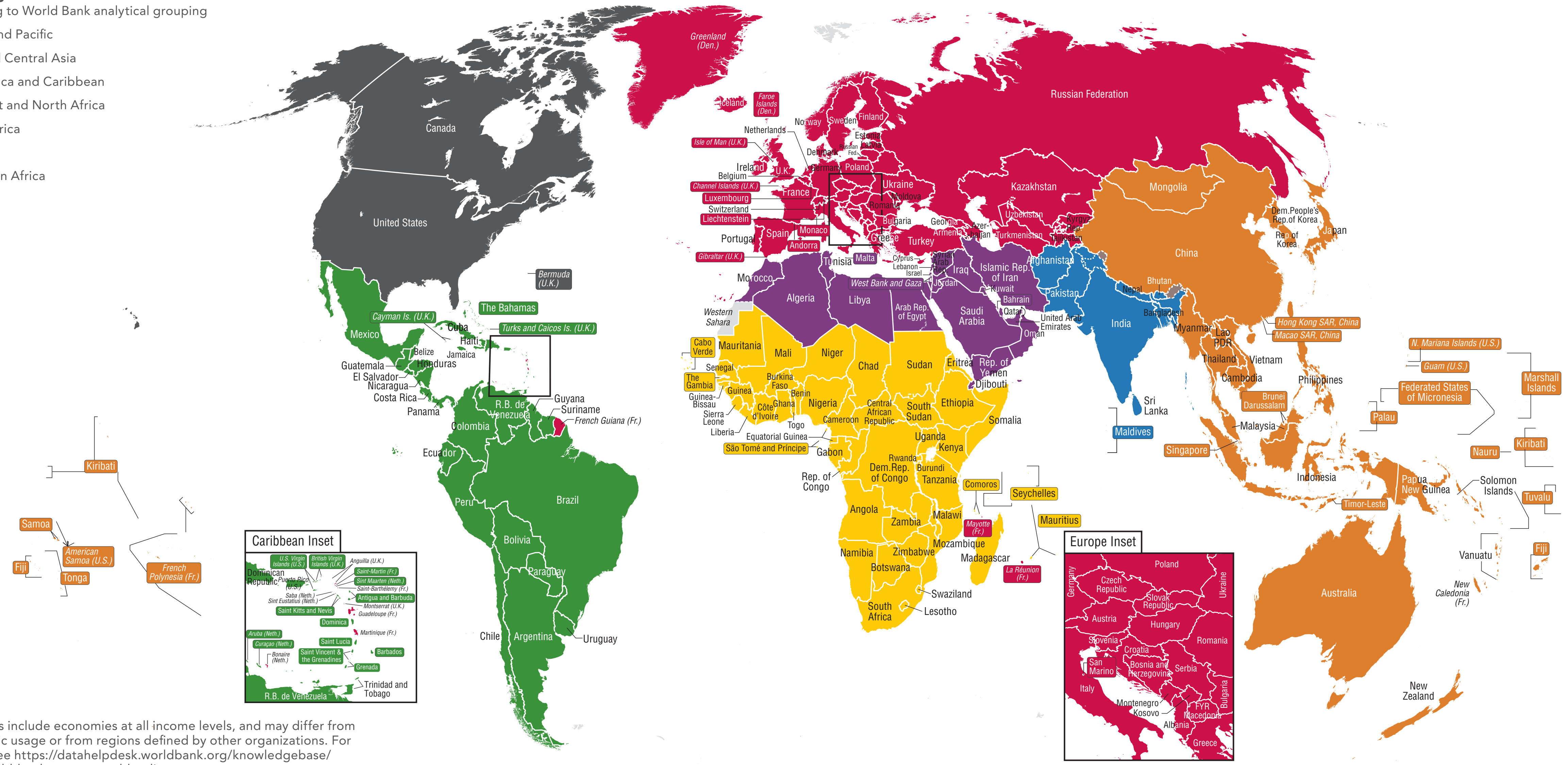

Note: These regions include economies at all income levels, and may differ from common geographic usage or from regions defined by other organizations. For more information see https://datahelpdesk.worldbank.org/knowledgebase/articles/906519-world-bank-country-and-lending-groups.

**Grenada**	Upper middle income
**Guatemala**	Lower middle income
**Guyana**	Upper middle income
**Haiti**	Low income
**Honduras**	Lower middle income
**Jamaica**	Upper middle income
**Mexico**	Upper middle income
**Nicaragua**	Lower middle income
**Panama**	Upper middle income
**Paraguay**	Upper middle income
**Peru**	Upper middle income
**Puerto Rico**	High income
**Sint Maarten**	High income
**St. Kitts and Nevis**	High income
**St. Lucia**	Upper middle income
**St. Martin**	High income
**St. Vincent and the Grenadines**	Upper middle income
**Suriname**	Upper middle income
**Trinidad and Tobago**	High income
**Turks and Caicos Islands**	High income
**Uruguay**	High income
**Venezuela, RB**	Upper middle income
**Virgin Islands (U.S.)**	High income
**Middle East and North Africa**	
**Algeria**	Upper middle income
**Bahrain**	High income
**Djibouti**	Lower middle income
**Egypt, Arab Rep.**	Lower middle income
**Iran, Islamic Rep.**	Upper middle income
**Iraq**	Upper middle income
**Israel**	High income
**Jordan**	Lower middle income
**Kuwait**	High income
**Lebanon**	Upper middle income
**Libya**	Upper middle income
**Malta**	High income
**Morocco**	Lower middle income
**Oman**	High income
**Qatar**	High income
**Saudi Arabia**	High income
**Syrian Arab Republic**	Lower middle income
**Tunisia**	Lower middle income
**United Arab Emirates**	High income
**West Bank and Gaza**	Lower middle income
**Yemen, Rep.**	Lower middle income
**North America**	
**Bermuda**	High income
**Canada**	High income
**United States**	High income
**South Asia**	
**Afghanistan**	Low income
**Bangladesh**	Lower middle income
**Bhutan**	Lower middle income
**India**	Lower middle income
**Maldives**	Upper middle income
**Nepal**	Low income
**Pakistan**	Lower middle income
**Sri Lanka**	Lower middle income
**Sub-Saharan Africa**	
**Angola**	Lower middle income
**Benin**	Low income
**Botswana**	Upper middle income
**Burkina Faso**	Low income
**Burundi**	Low income
**Cabo Verde**	Lower middle income
**Cameroon**	Lower middle income
**Central African Republic**	Low income
**Chad**	Low income
**Comoros**	Low income
**Congo, Dem. Rep.**	Low income
**Congo, Rep.**	Lower middle income
**Côte d'Ivoire**	Lower middle income
**Equatorial Guinea**	Upper middle income
**Eritrea**	Low income
**Ethiopia**	Low income
**Gabon**	Upper middle income
**Gambia, The**	Low income
**Ghana**	Lower middle income
**Guinea**	Low income
**Guinea-Bissau**	Low income
**Kenya**	Lower middle income
**Lesotho**	Lower middle income
**Liberia**	Low income
**Madagascar**	Low income
**Malawi**	Low income
**Mali**	Low income
**Mauritania**	Lower middle income
**Mauritius**	Upper middle income
**Mozambique**	Low income
**Namibia**	Upper middle income
**Niger**	Low income
**Nigeria**	Lower middle income
**Rwanda**	Low income
**São Tomé and Principe**	Lower middle income
**Senegal**	Low income
**Seychelles**	High income
**Sierra Leone**	Low income
**Somalia**	Low income
**South Africa**	Upper middle income
**South Sudan**	Low income
**Sudan**	Lower middle income
**Swaziland**	Lower middle income
**Tanzania**	Low income
**Togo**	Low income
**Uganda**	Low income
**Zambia**	Lower middle income
**Zimbabwe**	Low income
