# Supplementary material for: Global estimates of rehabilitation needs and disease burden in tracheal, bronchus, and lung cancer from 1990 to 2019 and projections to 2045 based on the global burden of disease study 2019
Source: Front Oncol. 2023 Jun 29;13:1152209. doi: 10.3389/fonc.2023.1152209 (PMC10344363; doi:10.3389/fonc.2023.1152209)
Supplement: Supplementary file 1 [file DataSheet_1.zip › Supplementary Material/Supplementary Material 3 BAPC model R code- Prevalence.pdf]

Supplementary Material 3

R codes: BAPC-predict- Prevalence

```
setwd('G:/GBD data/Data') ##Setting the work path

##Install and library R packages for prediction process and plotting
install.packages("caTools")
install.packages("cmprsk")
install.packages("fanplot")
install.packages("Epi")
install.packages("BAPC", repos = "http://R-Forge.R-project.org")
library(caTools)
library(Epi)
library(fanplot)
library(colorspace)
library(BAPC)
install.packages('sp')
install.packages("G:/GBD data/R_packages/INLA_22.05.07.zip", repos = NULL)
library(sp)
library(Matrix)
library(foreach)
library(parallel)
library(INLA)
library(nordpred)
library(tidyverse)
library(epitools)
library(ggplot2)
install.packages("Matrix")
library(Matrix)
install.packages("reshape")
library(reshape)
install.packages("data.table")
library(data.table)
install.packages("reshape2")
library(reshape2)
install.packages("tidyr")
library(tidyr)
library(tidyverse)
library(epitools)
library(ggplot2)

### Data Acquisition
EC <- read.csv('EC_predict.csv')##The Prevalence data downloaded from the GBD database
age_stand <- read.csv('std_population.csv')
```

```
#### Age stratification of Prevalence data needs
ages<- c("1-4 years", "5-9 years", "10-14 years", "15-19 years", "20-24 years", "25-29
years",
             "30-34 years", "35-39 years", "40-44 years", "45-49 years", "50-54 years", "55-
59 years",
             "60-64 years", "65-69 years", "70-74 years", "75-79 years", "80-84 years", "85-
89 years",
             "90-94 years", "95+ years")

#### Age stratification of the total population needs
##Population of GBD
ages_2<- c("Under 5", "5 to 9","10 to 14", "15 to 19","20 to 24", "25 to 29",
                "30 to 34", "35 to 39", "40 to 44", "45 to 49", "50 to 54", "55 to 59",
                "60 to 64", "65 to 69", "70 to 74", "75 to 79", "80 to 84", "85 to 89",
                "90 to 94", "95 plus")

##WHO population and standardized population
age_2_1<- c("0-4", "5-9", "10-14", "15-19", "20-24", "25-29",
                 "30-34", "35-39", "40-44", "45-49", "50-54", "55-59",
                 "60-64", "65-69", "70-74", "75-79", "80-84", "85-89",
                 "90-94", "95-99", "100+")

age_2_2<- c("<1 year","1 to 4", "5 to 9","10 to 14", "15 to 19","20 to 24", "25 to 29",
                 "30 to 34", "35 to 39", "40 to 44", "45 to 49", "50 to 54", "55 to 59",
                 "60 to 64", "65 to 69", "70 to 74", "75 to 79", "80 to 84", "85 to 89",
                 "90 to 94", "95 plus")

###### Age structure incorporated into the model
age_3<- c("0-4", "5-9", "10-14", "15-19", "20-24", "25-29",
              "30-34", "35-39", "40-44", "45-49", "50-54", "55-59",
              "60-64", "65-69", "70-74", "75-79", "80-84", "85-89",
              "90-94", "95+")

#Obtaining standardized population data
age_stand <- subset(age_stand,age %in% age_2_2)
wstand <- c(age_stand$std_population[1:2] %>% as.numeric() %>% sum(),
                 age_stand$std_population[3:21]                                        %>%
as.numeric())/sum(age_stand$std_population[1:21])

###Prevalence for Male and female
EC_Male_Prevalence <- subset(EC,age %in% ages &
                                          sex == 'Male' &
                                          metric == 'Number' &
```

```
                                          measure == 'Prevalence' &
                                          location== 'Global')[,c(3,4,7,8)]
EC_Male_Prevalence <- within(EC_Male_Prevalence,{
  age[age=="1-4 years"] <- "0-4"
  age[age=="5-9 years"]<-"5-9"
  age[age=="10-14 years"]<-"10-14"
  age[age=="15-19 years"]<-"15-19"
  age[age=="20-24 years"]<-"20-24"
  age[age=="25-29 years"]<-"25-29"
  age[age=="30-34 years"]<-"30-34"
  age[age=="35-39 years"]<-"35-39"
  age[age=="40-44 years"]<-"40-44"
  age[age=="45-49 years"]<-"45-49"
  age[age=="50-54 years"]<-"50-54"
  age[age=="55-59 years"]<-"55-59"
  age[age=="60-64 years"]<-"60-64"
  age[age=="65-69 years"]<-"65-69"
  age[age=="70-74 years"]<-"70-74"
  age[age=="75-79 years"]<-"75-79"
  age[age=="80-84 years"]<-"80-84"
  age[age=="85-89 years"]<-"85-89"
  age[age=="90-94 years"]<-"90-94"
  age[age=="95+ years"]<-"95+"})
EC_Male_Prevalence_n <- reshape2::dcast(data = EC_Male_Prevalence, year~age, value.var = "val")
rownames(EC_Male_Prevalence_n) <- EC_Male_Prevalence_n$year
EC_Male_Prevalence_n <- EC_Male_Prevalence_n[,-1]


library(tidyverse)
EC_Male_Prevalence_n <- apply(EC_Male_Prevalence_n, c(1,2), as.integer) %>% as.data.frame()
EC_Male_Prevalence_n <- apply(EC_Male_Prevalence_n, c(1,2), round) %>% as.data.frame()


EC_Female_Prevalence <- subset(EC,age %in% ages &
                                            sex == 'Female' &
                                            metric == 'Number' &
                                            measure == 'Prevalence' &
                                            location == 'Global')[,c(3,4,7,8)]
EC_Female_Prevalence <- within(EC_Female_Prevalence,{
  age[age=="1-4 years"] <- "0-4"
  age[age=="5-9 years"]<-"5-9"
  age[age=="10-14 years"]<-"10-14"
```

```
  age[age=="15-19 years"]<-"15-19"
  age[age=="20-24 years"]<-"20-24"
  age[age=="25-29 years"]<-"25-29"
  age[age=="30-34 years"]<-"30-34"
  age[age=="35-39 years"]<-"35-39"
  age[age=="40-44 years"]<-"40-44"
  age[age=="45-49 years"]<-"45-49"
  age[age=="50-54 years"]<-"50-54"
  age[age=="55-59 years"]<-"55-59"
  age[age=="60-64 years"]<-"60-64"
  age[age=="65-69 years"]<-"65-69"
  age[age=="70-74 years"]<-"70-74"
  age[age=="75-79 years"]<-"75-79"
  age[age=="80-84 years"]<-"80-84"
  age[age=="85-89 years"]<-"85-89"
  age[age=="90-94 years"]<-"90-94"
  age[age=="95+ years"]<-"95+"})
EC_Female_Prevalence_n <- reshape2::dcast(data = EC_Female_Prevalence, year~age,
value.var = "val")
rownames(EC_Female_Prevalence_n) <- EC_Female_Prevalence_n$year
EC_Female_Prevalence_n <- EC_Female_Prevalence_n[,-1]

EC_Female_Prevalence_n <- apply(EC_Female_Prevalence_n, c(1,2), as.integer) %>%
as.data.frame()
EC_Female_Prevalence_n <- apply(EC_Female_Prevalence_n, c(1,2), round) %>%
as.data.frame()

##### Get demographic data for 1990-2019
dirname <- dir("G:/GBD data/Data/GBD_Population")  ###Read files from a directory
folder
file <- paste0("G:/GBD data/Data/GBD_Population/",dirname)  ### Add the file with a
path
var_name <- c("location_name","sex_name","year_id","age_group_name","val")  ##Set up
the required variables

GBD_population  <-  as.data.frame(matrix(nrow=0,ncol=length(var_name)))
names(GBD_population)=var_name
for (a in file) {
  data <- fread(a) %>% select(var_name) %>%
    filter(age_group_name %in% ages_2)
  GBD_population <- rbind(GBD_population,data)
}  ###The purpose of the cycle is to read out the number of people in different age groups
from 1990-2019
GBD_population$sex_name[GBD_population$sex_name=='both'] <- 'Both'
```

```
GBD_population$sex_name[GBD_population$sex_name=='male'] <- 'Male'
GBD_population$sex_name[GBD_population$sex_name=='female'] <- 'Female'
GBD_population <- GBD_population[!duplicated(GBD_population),]
GBD_population <- within(GBD_population,{
  age_group_name[age_group_name=="Under 5"] <- "0-4"
  age_group_name[age_group_name=="5 to 9"]<-"5-9"
  age_group_name[age_group_name=="10 to 14"]<-"10-14"
  age_group_name[age_group_name=="15 to 19"]<-"15-19"
  age_group_name[age_group_name=="20 to 24"]<-"20-24"
  age_group_name[age_group_name=="25 to 29"]<-"25-29"
  age_group_name[age_group_name=="30 to 34"]<-"30-34"
  age_group_name[age_group_name=="35 to 39"]<-"35-39"
  age_group_name[age_group_name=="40 to 44"]<-"40-44"
  age_group_name[age_group_name=="45 to 49"]<-"45-49"
  age_group_name[age_group_name=="50 to 54"]<-"50-54"
  age_group_name[age_group_name=="55 to 59"]<-"55-59"
  age_group_name[age_group_name=="60 to 64"]<-"60-64"
  age_group_name[age_group_name=="65 to 69"]<-"65-69"
  age_group_name[age_group_name=="70 to 74"]<-"70-74"
  age_group_name[age_group_name=="75 to 79"]<-"75-79"
  age_group_name[age_group_name=="80 to 84"]<-"80-84"
  age_group_name[age_group_name=="85 to 89"]<-"85-89"
  age_group_name[age_group_name=="90 to 94"]<-"90-94"
  age_group_name[age_group_name=="95 plus"]<-"95+"})
GBD_population_Male <- subset(GBD_population,location_name=='Global' & sex_name
== 'Male')
GBD_population_Female <- subset(GBD_population,location_name=='Global' & sex_name
== 'Female')


##Crowd projection data collation
prediction_var_name <- c("Location","Time","AgeGrp","PopMale","PopFemale")
WHO_population_prediction <- fread('WPP2019_PopulationByAgeSex_Medium.csv') %>%
  select(prediction_var_name) %>%
  filter(AgeGrp %in% age_2_1 & Time %in% 2020:2045)
names(WHO_population_prediction) <- prediction_var_name
Location_Global <- WHO_population_prediction %>% subset(Location %in% c("World")) %>%
  group_by(Time,AgeGrp) %>%
  summarize(PopMale=sum(PopMale), PopFemale=sum(PopFemale))
Location_Global$Location <- 'World'
Location_Global <- Location_Global[,c(5,1:4)]
WHO_age95 <- Location_Global %>% subset(AgeGrp %in% c("95-99","100+")) %>%
  group_by(Location,Time) %>%
  summarize(PopMale=sum(PopMale), PopFemale=sum(PopFemale))
```

```
WHO_age95$AgeGrp <- '95+'
WHO_age95 <- WHO_age95[,c(1:2,5,3:4)]
WHO_population_prediction <- subset(Location_Global, AgeGrp %in% age_3[-c(20)])
WHO_population_prediction <- rbind(WHO_population_prediction,WHO_age95)
WHO_population_prediction$PopMale <- WHO_population_prediction$PopMale*1000
WHO_population_prediction$PopFemale <-
WHO_population_prediction$PopFemale*1000
WHO_Global_Male <- WHO_population_prediction[,c(1:4)]
WHO_Global_Male$sex_name <- 'Male'
WHO_Global_Male <- WHO_Global_Male[,c(1,5,2:4)]
names(WHO_Global_Male)[1] <- "location_name"
names(WHO_Global_Male)[3] <- "year_id"
names(WHO_Global_Male)[4] <- "age_group_name"
names(WHO_Global_Male)[5] <- "val"
WHO_Global_Female <- WHO_population_prediction[,c(1:3,5)]
WHO_Global_Female$sex_name <- 'Female'
WHO_Global_Female <- WHO_Global_Female[,c(1,5,2:4)]
names(WHO_Global_Female)[1] <- "location_name"
names(WHO_Global_Female)[3] <- "year_id"
names(WHO_Global_Female)[4] <- "age_group_name"
names(WHO_Global_Female)[5] <- "val"

## Combining data from 1990-2019 and 2020-2045
##Resize population data to the desired format
GBD_Global_Male <- rbind(GBD_population_Male,WHO_Global_Male)
GBD_Global_Female <- rbind(GBD_population_Female,WHO_Global_Female)


GBD_Global_Male_n <- reshape2::dcast(data = GBD_Global_Male, year_id ~
age_group_name,value.var = c("val"))
GBD_Global_Male_n <- as.data.frame(GBD_Global_Male_n)
rownames(GBD_Global_Male_n) <- GBD_Global_Male_n$year_id
GBD_Global_Male_n <- GBD_Global_Male_n[,-1]
GBD_Global_Male_n <- GBD_Global_Male_n[,c(1,10,2:9,11:20)]####Age order from
youngest to oldest

GBD_Global_Female_n <- reshape2::dcast(data = GBD_Global_Female, year_id ~
age_group_name,value.var = c("val"))
GBD_Global_Female_n <- as.data.frame(GBD_Global_Female_n)
rownames(GBD_Global_Female_n) <- GBD_Global_Female_n$year_id
GBD_Global_Female_n <- GBD_Global_Female_n[,-1]
GBD_Global_Female_n <- GBD_Global_Female_n[,c(1,10,2:9,11:20)]####Age order from
youngest to oldest
```

```
GBD_Global_Male_n <- apply(GBD_Global_Male_n, c(1,2), as.numeric) %>% as.data.frame()
GBD_Global_Female_n <- apply(GBD_Global_Female_n, c(1,2), as.numeric) %>%
as.data.frame()
GBD_Global_Male_n <- apply(GBD_Global_Male_n, c(1,2), round) %>% as.data.frame()
GBD_Global_Female_n <- apply(GBD_Global_Female_n, c(1,2), round) %>% as.data.frame()

GBD_Global_Both_n <- GBD_Global_Female_n + GBD_Global_Male_n

##Additional years with no data for the Prevalence
EC_pro <- matrix(data = NA, nrow = 2045-2019, ncol = ncol(GBD_Global_Male_n)) %>%
as.data.frame()
rownames(EC_pro) <- seq(2020,2045,1)
colnames(EC_pro) <-  names(EC_Male_Prevalence_n)

EC_Male_Prevalence_n <- EC_Male_Prevalence_n[,c(1,10,2:9,11:20)]####Age order from
youngest to oldest
EC_Female_Prevalence_n <- EC_Female_Prevalence_n[,c(1,10,2:9,11:20)]####Age order
from youngest to oldest
EC_Male_Prevalence_n  <- rbind(EC_Male_Prevalence_n , EC_pro)##Male data merge
EC_Female_Prevalence_n  <- rbind(EC_Female_Prevalence_n , EC_pro)##Female data
merge


####### Applying the BAPC model for forecasting
Male_esoph <- APCList(EC_Male_Prevalence_n, GBD_Global_Male_n, gf = 5)
Male_bapc_result <- BAPC(Male_esoph, predict = list(npredict = 26, retro = T),
                         secondDiff = FALSE, stdweight = wstand, verbose = F)

Female_esoph <- APCList(EC_Female_Prevalence_n, GBD_Global_Female_n, gf = 5)
Female_bapc_result <- BAPC(Female_esoph, predict = list(npredict = 26, retro = T),
                           secondDiff = FALSE, stdweight = wstand, verbose = F)

##### Number of Prevalence by age group
Male_proj <- agespec.proj(x = Male_bapc_result) %>% as.data.frame()   ### Find a
predicted population
Male_proj_mean <- Male_proj[,colnames(Male_proj) %like% 'mean']
colnames(Male_proj_mean) <- age_3

Female_proj <- agespec.proj(x = Female_bapc_result) %>% as.data.frame()   ### Find a
predicted population
Female_proj_mean <- Female_proj[,colnames(Female_proj) %like% 'mean']
colnames(Female_proj_mean) <- age_3

Both_proj_mean <- Female_proj_mean+ Male_proj_mean
```

```
##### Rates for different age groups
Male_rate <- agespec.rate(x = Male_bapc_result) %>% as.data.frame()
Male_rate_mean <- Male_rate[,colnames(Male_rate) %like% 'mean']*100000
colnames(Male_rate_mean) <- age_3

Female_rate <- agespec.rate(x = Female_bapc_result) %>% as.data.frame()
Female_rate_mean <- Female_rate[,colnames(Female_rate) %like% 'mean']*100000
colnames(Female_rate_mean) <- age_3

Both_rate_mean <- Both_proj_mean/GBD_Global_Both_n*100000


#####Calculation of age-corrected rates
Male_ASR <- agestd.rate(x = Male_bapc_result) %>% as.data.frame()
Male_ASR$mean <- Male_ASR$mean*100000
Male_ASR$year <- rownames(Male_ASR)

Female_ASR <- agestd.rate(x =Female_bapc_result) %>% as.data.frame()
Female_ASR$mean <- Female_ASR$mean*100000
Female_ASR$year <- rownames(Female_ASR)

year_index <- 1990:2045
Both_ASR <- matrix(nrow = 0, ncol = 2) %>% as.data.frame()
names(Both_ASR) <- c("crude.rate","adj.rate")
#i=1
for (i in 1:(2045-1989)) {
  asr = ageadjust.direct(count = Both_proj_mean[i,], pop = GBD_Global_Both_n[i,],
                         stdpop = wstand)
  Both_ASR[i,1:2] <- round(asr[1:2]*10^5,2)
}
Both_ASR$year <- 1990:2045


#####Total number of cases
Male_sum_year <- apply(Male_proj_mean, 1, sum) %>% as.data.frame()
colnames(Male_sum_year) <- 'number'
Male_sum_year$year <- rownames(Male_sum_year)


Female_sum_year <- apply(Female_proj_mean, 1, sum) %>% as.data.frame()
colnames(Female_sum_year) <- 'number'
Female_sum_year$year <- rownames(Female_sum_year)
```

```
Male_sum_year <- apply(Male_sum_year, 2, as.numeric) %>% as.data.frame()
Female_sum_year <- apply(Female_sum_year, 2, as.numeric) %>% as.data.frame()
Both_proj_mean <- Female_proj_mean + Male_proj_mean
Both_sum_year <- Male_sum_year + Female_sum_year
Both_sum_year$year <- 1990:2045


#Mapping of male projections
plotBAPC(Male_bapc_result, scale=10^5, type = 'ageStdProj', showdata = TRUE)

#Mapping of female projections
plotBAPC(Female_bapc_result, scale=10^5, type = 'ageStdRate', showdata = TRUE)

#Prediction of Both genders
EC_Both_Prevalence_n <- EC_Female_Prevalence_n + EC_Male_Prevalence_n
Both_esoph <- APCList(EC_Both_Prevalence_n, GBD_Global_Both_n, gf = 5)
Both_bapc_result <- BAPC(Both_esoph, predict = list(npredict = 26, retro = T),
                         secondDiff = FALSE, stdweight = wstand, verbose = F)
Both_ASR <- agestd.rate(x = Both_bapc_result) %>% as.data.frame()
Both_ASR$mean <- Both_ASR$mean*100000
Both_ASR$year <- rownames(Both_ASR)

#Mapping of Both projections
plotBAPC(Both_bapc_result, scale=10^5, type = 'ageStdRate', showdata = TRUE)
```
