# Supplementary material for: Global estimates of rehabilitation needs and disease burden in tracheal, bronchus, and lung cancer from 1990 to 2019 and projections to 2045 based on the global burden of disease study 2019
Source: Front Oncol. 2023 Jun 29;13:1152209. doi: 10.3389/fonc.2023.1152209 (PMC10344363; doi:10.3389/fonc.2023.1152209)
Supplement: Supplementary file 1 [file DataSheet_1.zip › Supplementary Material/Supplementary Material 3 BAPC model.pdf]

Bayesian age-period-cohort (BAPC) model

Bayesian age-period-cohort (BAPC) model is based on the theory that there is a correlation between morbidity or mortality and age structure and population size. The BAPC model turned out to be the most appropriate statistical methods of projecting the cancer burden compared with generalized additive model, smooth spline model, Nordpred model, Joinpoint model, and Poisson regression, especially for short-term projections[1-3] .

The rationalities of the Bayesian APC model have been previously described [4]. Briefly, since the expectation that effects adjacent in time might be similar, the second-order random walk (RW2) model with inverse-gamma prior distribution was used for age, period and cohort effects. RW2 assumes an independent mean-zero normal distribution of the second differences of all time effects. This is a natural target for smoothing since the second differences in APC models are identifiable. Consider the age effects, for which the RW2 prior is identified as follows:

$$f(a|\kappa_a))\infty\kappa_a\frac{I-2}{2}\exp\left(-\frac{\kappa_a}{2}\sum_{i=3}^{I}(a_i-2a_{i-1}+a_{i-2})^2\right)=\kappa_a\frac{I-2}{2}\exp\left(-\frac{1}{2}a^T Qa\right)$$

$$Q=\kappa_a\begin{bmatrix}1 & 2 & 1 & & & & \\ -2 & 5 & -4 & 1 & & & \\ 1 & -4 & 6 & -4 & 1 & & \\ & O & O & O & O & O & \\ & & 1 & -4 & 6 & -4 & 1 \\ & & & 1 & -4 & 5 & -2 \\ & & & & 1 & -2 & 1\end{bmatrix}$$

where i denotes the age index that ranges from 1 to I = 17 in this study, because we projected the cancer incidence of people aged 0 to 84, and age was divided into 17 groups. Moreover, $\kappa\alpha - 1$ denotes the variance parameter. Note that Q is rank deficient. To complete the RW2 model specification, we use the usual conjugate hyperprior for precision, $\kappa\alpha \sim \text{Gamma}(\alpha, \lambda)$. This leads to the full conditional $\kappa\alpha|\alpha \sim \text{Gamma}(\alpha + 0.5\ \text{rank}(Q), \lambda + 0.5\alpha'Q\alpha)$, which may be directly simulated [5]. In this study, we used the parameter values $\alpha = 0.5$, 1, and 1 and $\lambda = 0.0005$, 0.00005, and 0.00005 for age, period, and cohort effects, respectively. The World-2000 population was used to standardize the KC incidence rates. To ensure the smoothness of predictions, countries or territories that experienced a striking fluctuation in KC case numbers within a small time interval were excluded.

Briefly, the age-period-cohort model, $\eta ij = \log(\lambda ij) = \mu + \alpha i + \beta j + \gamma k$, was fitted as a log-linear Poisson model, in which λij indicated the number of cases, μ was the intercept, and αi, βj, γk were age, birth cohort and the period in which the event occurred, respectively.24 i ($1 \leq i \leq I$) indicated

age group at time j ($1 \leq j \leq J$), and the birth cohort index k, $k = j + M(I - i)$, depended on the age group and period index, as well as the width of age groups and period intervals, where M indicated the width of age groups (5 in this analysis) divided by the period intervals. BAPC model (the BAPC package via software R) fitted with Integrated Nested Laplace Approximation (the INLA package via software R) was used to project breast cancer burden by sex until 2045, based on the hypothesis of inverse-gamma prior distribution of the GBD 2019 data we retrieved, including age, period, and cohort effects (via the second-order random walk model) to adjust for excessive dispersion.

Reference:

1. Yu J, Yang X, He W, Ye W. Burden of pancreatic cancer along with attributable risk factors in Europe between 1990 and 2019, and projections until 2039. Int J Cancer. 2021; 149(5): 993- 1001. doi:10.1002/ijc.33617

2. Knoll M, Furkel J, Debus J, Abdollahi A, Karch A, Stock C. An R package for an integrated evaluation of statistical approaches to cancer incidence projection. BMC Med Res Methodol. 2020; 20(1): 257. doi:10.1186/s12874-020-01133-5

3. Du Z, Chen W, Xia Q, Shi O, Chen Q. Trends and projections of kidney cancer incidence at the global and national levels, 1990-2030: a Bayesian age-period-cohort modeling study. Biomark Res. 2020; 8: 16. doi:10.1186/s40364-020-00195-3

4. Riebler A, Held L. Projecting the future burden of cancer: Bayesian age-period-cohort analysis with integrated nested Laplace approximations. Biom J. 2017;59:531–549. doi: 10.1002/bimj.201500263

5. Liu Z, Jiang Y, Fang Q, Yuan H, Cai N, Suo C, et al. Future of cancer incidence in Shanghai, China: predicting the burden upon the ageing population. Cancer Epidemiol. 2019;60:8–15. doi: 10.1016/j.canep.2019.03.004
