# Supplementary material for: Global estimates of rehabilitation needs and disease burden in tracheal, bronchus, and lung cancer from 1990 to 2019 and projections to 2045 based on the global burden of disease study 2019
Source: Front Oncol. 2023 Jun 29;13:1152209. doi: 10.3389/fonc.2023.1152209 (PMC10344363; doi:10.3389/fonc.2023.1152209)
Supplement: Supplementary file 1 [file DataSheet_1.zip › Supplementary Material/Supplementary Material 4 ARIMA model analysis.pdf]

ARIMA model analysis

The Autoregressive Integrated Moving Average (ARIMA) model is an investigative, data-oriented method, allowing the user to adjust the robustly oriented procedures that change over time by forecasting the scenarios future condition based on current situations [1, 2]. We choose to use modest time-series methods such as the ARIMA model to forecast the ASR of the upcoming 26 year until the end of 2045 in this research. To fit the model, we consider the model as ARIMA (p, d, q), where p represents the autoregressive term, d denotes the differencing order, and q indicates the moving averages term. Since the ARIMA model is the amalgamation of Autoregressive (AR) and Moving Average (MA) terms, we believe that it fits sound to the nature of the data and delivers good short-term forecasting. Parameters (p, d, q) are recognized by Autocorrelation function (ACF) and Partial Autocorrelation Function (PACF) for assessing the model fit. In addition, ARIMA (p,d,q) is chosen based on the Akaike information criterion (AIC), a goodness of fit test where the model with minimum AIC is considered here. We use R package tseries, and forecast to fit the ARIMA model. We run the ARIMA model through auto.arima function under the package forecast.

1. Dyer, O., *Covid-19: Remdesivir has little or no impact on survival, WHO trial shows.* BMJ, 2020. **371**: p. m4057.
2. Papastefanopoulos, V., P. Linardatos, and S. Kotsiantis, *COVID-19: A Comparison of Time Series Methods to Forecast Percentage of Active Cases per Population.* Applied Sciences, 2020. **10**(11): p. 3880.
