# Supplementary material for: Global estimates of rehabilitation needs and disease burden in tracheal, bronchus, and lung cancer from 1990 to 2019 and projections to 2045 based on the global burden of disease study 2019
Source: Front Oncol. 2023 Jun 29;13:1152209. doi: 10.3389/fonc.2023.1152209 (PMC10344363; doi:10.3389/fonc.2023.1152209)
Supplement: Supplementary file 1 [file DataSheet_1.zip › Supplementary Material/Supplementary Material 4 ARIMA model R code- YLDs.pdf]

Supplementary Material 4

R codes: ARIMA -predict- YLDs

```
###The number of YLDs prediction code
#Both
setwd('G:/GBD data/Data/ARIMA')
YLDs <- read.csv('YB.csv')
YLDsseris<-ts(YLDs,start = c(1990))
library(forecast)
auto.arima(YLDs,ic = "bic")
YLDsarima<-arima(YLDsseris,order = c(0,2,0))##"c=(0,2,0)" is based on the result of the "ARIMA(0,2,0)"
YLDsserisforecast<-forecast(YLDsarima,h = 26)
YLDsserisforecast
plot(YLDsserisforecast)
plot(YLDsserisforecast,col="black",pch=25,bg="yellow",lwd=2,main=2,asp=0,cex=1.2,lty=2)
acf(YLDsserisforecast$residuals,lag.max = 20)
Box.test(YLDsserisforecast$residuals,lag = 20,type = "Ljung-Box")
write.csv(YLDsserisforecast,'Results for YBforecast.csv')


#Male
setwd('G:/GBD data/Data/ARIMA')
YLDs <- read.csv('YM.csv')
YLDsseris<-ts(YLDs,start = c(1990))
library(forecast)
auto.arima(YLDs,ic = "bic")
YLDsarima<-arima(YLDsseris,order = c(0,2,1))#auto.arima(YLDs,ic = "bic")??Series: YLDs??ARIMA(0,2,1)
YLDsserisforecast<-forecast(YLDsarima,h = 26)
YLDsserisforecast
plot(YLDsserisforecast)
plot(YLDsserisforecast,col="black",pch=25,bg="yellow",lwd=2,main=2,asp=0,cex=1.2,lty=2)
acf(YLDsserisforecast$residuals,lag.max = 20)
Box.test(YLDsserisforecast$residuals,lag = 20,type = "Ljung-Box")
write.csv(YLDsserisforecast,'Results for YMforecast.csv')

#Female
setwd('G:/GBD data/Data/ARIMA')
YLDs <- read.csv('YF.csv')
YLDsseris<-ts(YLDs,start = c(1990))
library(forecast)
auto.arima(YLDs,ic = "bic")
```

```
YLDsarima<-arima(YLDsseris,order = c(0,2,0))
YLDsserisforecast<-forecast(YLDsarima,h = 26)
YLDsserisforecast
plot(YLDsserisforecast)
plot(YLDsserisforecast,col="black",pch=25,bg="yellow",lwd=2,main=2,asp=0,cex=1.2,lty=
2)
acf(YLDsserisforecast$residuals,lag.max = 20)
Box.test(YLDsserisforecast$residuals,lag = 20,type = "Ljung-Box")
write.csv(YLDsserisforecast,'Results for YFforecast.csv')
```
