# Supplementary material for: Global estimates of rehabilitation needs and disease burden in tracheal, bronchus, and lung cancer from 1990 to 2019 and projections to 2045 based on the global burden of disease study 2019
Source: Front Oncol. 2023 Jun 29;13:1152209. doi: 10.3389/fonc.2023.1152209 (PMC10344363; doi:10.3389/fonc.2023.1152209)
Supplement: Supplementary file 1 [file DataSheet_1.zip › Supplementary Material/Supplementary Material 5.pdf]

Supplementary Material 5

1.The codes that were used for generating a part of the table 1，and it is also necessary to manually integrate the data of Prevalence and YLDs together into Table 1.

Part 1：Prevalence

```
setwd('G:/GBD data/XM GBD mode/GBD_Table') ##Setting the work path
## Retrieve the required R package
library(dplyr)
library(flextable)
library(officer)

EC <- read.csv('region.csv',header = T)   ## Retrieve data downloaded from the GBD database
order <- read.csv('order 1.csv',header = F)
EC$location <- factor(EC$location,
                      levels=order$V1,
                      ordered=TRUE)

## 1990 Number of Prevalence
EC_1990 <- subset(EC,EC$year==1990 &
                  EC$age=='All ages' &
                  EC$metric== 'Number' &
                  EC$measure=='Prevalence'&
                  sex=='Female')
EC_1990 <- subset(EC,EC$year==1990 &
                  EC$age=='All ages' &
                  EC$metric== 'Number' &
                  EC$measure=='Prevalence'&
```

```
                         sex=='Male')

EC_1990 <- EC_1990[,c(2,8,9,10)]   ### Take only the required variables: the region and the corresponding values
EC_1990$val <- round(EC_1990$val,0)   ###Rounding
EC_1990$lower <- round(EC_1990$lower,0)###Rounding
EC_1990$upper <- round(EC_1990$upper,0) ###Rounding
EC_1990$Num_1990 <- paste(EC_1990$lower,EC_1990$upper,sep = '-') ## Use - to connect 95% of the upper and lower values of the UI
EC_1990$Num_1990 <- paste(EC_1990$Num_1990,')',sep = '')   ##95% UI with brackets before and after
EC_1990$Num_1990 <- paste('(',EC_1990$Num_1990,sep = '')   ##95% UI with brackets before and after
EC_1990$Num_1990 <- paste(EC_1990$val,EC_1990$Num_1990,sep = '') ##Data and 95% of the UI are connected with the space bar

## 2019 Number of Prevalence
EC_2019 <- subset(EC,EC$year==2019 &
                         EC$age=='All ages' &
                         EC$metric== 'Number' &
                         EC$measure=='Prevalence'&
                         sex=='Female')
EC_2019 <- subset(EC,EC$year==2019 &
                         EC$age=='All ages' &
                         EC$metric== 'Number' &
                         EC$measure=='Prevalence'&
                         sex=='Male')

EC_2019 <- EC_2019[,c(2,8,9,10)]   ### Take only the required variables: the
```

```
region and the corresponding values
EC_2019$val <- round(EC_2019$val,0)    ###Rounding
EC_2019$lower <- round(EC_2019$lower,0)###Rounding
EC_2019$upper <- round(EC_2019$upper,0) ###Rounding
EC_2019$Num_2019 <- paste(EC_2019$lower,EC_2019$upper,sep = '-') ## Use -
to connect 95% of the upper and lower values of the UI
EC_2019$Num_2019 <- paste(EC_2019$Num_2019,')',sep = '')   ##95% UI with
brackets before and after
EC_2019$Num_2019 <- paste('(',EC_2019$Num_2019,sep = '')   ##95% UI with
brackets before and after
EC_2019$Num_2019 <- paste(EC_2019$val,EC_2019$Num_2019,sep = '') ##Data
and 95% of the UI are connected with the space bar

## 1990 ASR
ASR_1990 <- subset(EC,EC$year==1990 &
                    EC$age=='Age-standardized' &
                    EC$metric== 'Rate' &
                    EC$measure=='Prevalence'&
                    sex=='Female')
ASR_1990 <- subset(EC,EC$year==1990 &
                     EC$age=='Age-standardized' &
                     EC$metric== 'Rate' &
                     EC$measure=='Prevalence'&
                     sex=='Male')

ASR_1990 <- ASR_1990[,c(2,8,9,10)]   ### Take only the required variables: the
region and the corresponding values
ASR_1990$val <- round(ASR_1990$val,2)    ###Rounding
ASR_1990$lower <- round(ASR_1990$lower,2)###Rounding
```

```
ASR_1990$upper <- round(ASR_1990$upper,2) ###Rounding
ASR_1990$ASR_1990 <- paste(ASR_1990$lower,ASR_1990$upper,sep = '-') ## Use - to connect 95% of the upper and lower values of the UI
ASR_1990$ASR_1990 <- paste(ASR_1990$ASR_1990,')',sep = '')    ##95% UI with brackets before and after
ASR_1990$ASR_1990 <- paste('(',ASR_1990$ASR_1990,sep = '')    ##95% UI with brackets before and after
ASR_1990$ASR_1990 <- paste(ASR_1990$val,ASR_1990$ASR_1990,sep = '') ##Data and 95% of the UI are connected with the space bar

## 2019 ASR
ASR_2019 <- subset(EC,EC$year==2019 &
                   EC$age=='Age-standardized' &
                   EC$metric== 'Rate' &
                   EC$measure=='Prevalence'&
                   sex=='Female')
ASR_2019 <- subset(EC,EC$year==2019 &
                   EC$age=='Age-standardized' &
                   EC$metric== 'Rate' &
                   EC$measure=='Prevalence'&
                   sex=='Male')

ASR_2019 <- ASR_2019[,c(2,8,9,10)]
ASR_2019$val <- round(ASR_2019$val,2)
ASR_2019$lower <- round(ASR_2019$lower,2)
ASR_2019$upper <- round(ASR_2019$upper,2)
ASR_2019$ASR_2019 <- paste(ASR_2019$lower,ASR_2019$upper,sep = '-')
ASR_2019$ASR_2019 <- paste(ASR_2019$ASR_2019,')',sep = '')
ASR_2019$ASR_2019 <- paste('(',ASR_2019$ASR_2019,sep = '')
```

```
ASR_2019$ASR_2019 <- paste(ASR_2019$val,ASR_2019$ASR_2019,sep = '')

##### Calculating EAPC
EAPC <- subset(EC, EC$age=='Age-standardized' &
                    EC$metric== 'Rate' &
                    EC$measure=='Prevalence'&
                    sex=='Female')
EAPC <- subset(EC, EC$age=='Age-standardized' &
                    EC$metric== 'Rate' &
                    EC$measure=='Prevalence'&
                    sex=='Male')

EAPC <- EAPC[,c(2,7,8)] ##Get the region, year and the corresponding value

country <- EC_1990$location
EAPC_cal <-
data.frame(location=country,EAPC=rep(0,times=8),UCI=rep(0,times=8),LCI=rep(0
,times=8))
for (i in 1:8){   ###A total of 8 regions, so 8 cycles
  country_cal <- as.character(EAPC_cal[i,1]) ### Take the corresponding areas in
turn
  a <- subset(EAPC, EAPC$location==country_cal)   ##Take a subset of data from
the corresponding region
  a$y <- log(a$val)   ##Calculate the y-value according to the EAPC calculation
method
  mod_simp_reg<-lm(y~year,data=a) ##Do the linear regression equation according
to the EAPC calculation method
  estimate <- (exp(summary(mod_simp_reg)[["coefficients"]][2,1])-1)*100 ##The
EAPC is calculated by taking the beta value of the equation according to the EAPC
```

```
calculation method
   low <- (exp(summary(mod_simp_reg)[["coefficients"]][2,1]-1.96*summary(mod_simp_reg)[["coefficients"]][2,2])-1)*100
   ### Calculate the upper limit of the 95% confidence interval for EAPC
   high <- (exp(summary(mod_simp_reg)[["coefficients"]][2,1]+1.96*summary(mod_simp_reg)[["coefficients"]][2,2])-1)*100
   ### Calculate the lower limit of the 95% confidence interval for EAPC
   EAPC_cal[i,2] <- estimate
   EAPC_cal[i,4] <- low
   EAPC_cal[i,3] <- high
}

EAPC_cal$EAPC <- round(EAPC_cal$EAPC,2)    ##Retain 2 decimal places
EAPC_cal$UCI <- round(EAPC_cal$UCI,2)
EAPC_cal$LCI <- round(EAPC_cal$LCI,2)
EAPC_cal$EAPC_CI <- paste(EAPC_cal$LCI,EAPC_cal$UCI,sep = '-')
EAPC_cal$EAPC_CI <- paste(EAPC_cal$EAPC_CI,')',sep = '')
EAPC_cal$EAPC_CI <- paste('(',EAPC_cal$EAPC_CI,sep = '')
EAPC_cal$EAPC_CI <- paste(paste0(EAPC_cal$EAPC,'%'),EAPC_cal$EAPC_CI,sep = '')

###Change rate of prevalence
Prevalence_change <- read.csv('region-change.csv')
EC_change <- subset(Prevalence_change,
                    age=='All ages' &
                    metric== 'Rate' &
                    measure=='Prevalence'&
                    sex=='Female')
```

```
EC_change <- subset(Prevalence_change,
                            age=='All ages' &
                              metric== 'Rate' &
                              measure=='Prevalence'&
                              sex=='Male')

EC_change <- EC_change[,c(2,9,10,11)]
EC_change$val <- round(EC_change$val,2)
EC_change$lower <- round(EC_change$lower,2)
EC_change$upper <- round(EC_change$upper,2)
EC_change$Num_change <- paste(EC_change$lower,EC_change$upper,sep = '-')
EC_change$Num_change <- paste(EC_change$Num_change,')',sep = '')
EC_change$Num_change <- paste('(',EC_change$Num_change,sep = '')
EC_change$Num_change <-
paste(paste0(EC_change$val,'%'),EC_change$Num_change,sep = '')

# Integration Data
data <- flextable(Prevalence, col_keys = names(Prevalence))
data <- valign(data, valign = "center", part= "header")##Use the valign function to modify whether a table element is centered vertically, top-aligned or bottom-aligned
data <- align(data, align = "center", part= "all")##Use the align function to modify the alignment of table elements in the horizontal direction
data <- font(data, fontname = "Times New Roman", part = "all")##Use the font function to modify the font of elements in a table
data <- hline_top(data, border = fp_border(color="black", width = 1.5), part = "header") ##Use the hline_top function to modify the top line of the trilinear table, mainly in terms of color and line thickness
data <- hline_bottom(data, border = fp_border(color="black", width = 1.5), part = "body")##Use the hline_bottom function to make changes to the bottom line of the
```

```
trilinear table
data <- hline(data, i=1, border = fp_border(color="black", width =1), part = "header")##Use the hline function to make changes to the line in the middle of the three-line table
data <- set_caption(data, "Table 1-1.Prevalence of Tracheal, Bronchus, and Lung Cancer in 1990 and 2019 for both sexs in global and regions,with EAPC from 1990 and 2019.", autonum = 1, style = "Table Caption")##Use the function set_caption to set the title of the three-line table
data <- autofit(data) ##Automatic adjustment of the generated trilinear table using the autofit function


save_as_docx(data, path = "Table 1-1. Prevalence of Tracheal, Bronchus, and Lung Cancer in 1990 and 2019 for both sex in global and regions, with EAPC from 1990 and 2019.docx") ##Use the save_as_docx function to generate the three-line table object exported to Word documents


write.csv(Prevalence,'Results for Prevalence nation.csv')##Exporting integrated data
```

Part 2： YLDs

```
setwd('G:/GBD data/XM GBD mode/GBD_Table') ##Setting the work path
## Retrieve the required R package
library(dplyr)
library(flextable)
library(officer)
EC <- read.csv('region.csv',header = T)   ## Retrieve data downloaded from the GBD database
order <- read.csv('order 1.csv',header = F)
EC$location <- factor(EC$location,
                                  levels=order$V1,
                                  ordered=TRUE)

## 1990 Number of YLDs
EC_1990 <- subset(EC,EC$year==1990 &
                       EC$age=='All ages' &
```

```
                          EC$metric== 'Number' &
                          EC$measure=='YLDs (Years Lived with Disability)'&
                          sex=='Female')
EC_1990 <- subset(EC,EC$year==1990 &
                          EC$age=='All ages' &
                          EC$metric== 'Number' &
                          EC$measure=='YLDs (Years Lived with Disability)'&
                          sex=='Male')

EC_1990 <- EC_1990[,c(2,8,9,10)]   ### Take only the required variables: the region and the corresponding values
EC_1990$val <- round(EC_1990$val,0)   ###Rounding
EC_1990$lower <- round(EC_1990$lower,0)###Rounding
EC_1990$upper <- round(EC_1990$upper,0) ###Rounding
EC_1990$Num_1990 <- paste(EC_1990$lower,EC_1990$upper,sep = '-') ## Use - to connect 95% of the upper and lower values of the UI
EC_1990$Num_1990 <- paste(EC_1990$Num_1990,')',sep = '')   ##95% UI with brackets before and after
EC_1990$Num_1990 <- paste('(',EC_1990$Num_1990,sep = '')   ##95% UI with brackets before and after
EC_1990$Num_1990 <- paste(EC_1990$val,EC_1990$Num_1990,sep = '') ##Data and 95% of the UI are connected with the space bar

## 2019 Number of YLDs
EC_2019 <- subset(EC,EC$year==2019 &
                          EC$age=='All ages' &
                          EC$metric== 'Number' &
                          EC$measure=='YLDs (Years Lived with Disability)'&
                          sex=='Female')
EC_2019 <- subset(EC,EC$year==2019 &
                          EC$age=='All ages' &
                          EC$metric== 'Number' &
                          EC$measure=='YLDs (Years Lived with Disability)'&
                          sex=='Male')

EC_2019 <- EC_2019[,c(2,8,9,10)]   ### Take only the required variables: the region and the corresponding values
EC_2019$val <- round(EC_2019$val,0)   ###Rounding
EC_2019$lower <- round(EC_2019$lower,0)###Rounding
EC_2019$upper <- round(EC_2019$upper,0) ###Rounding
EC_2019$Num_2019 <- paste(EC_2019$lower,EC_2019$upper,sep = '-') ## Use - to connect 95% of the upper and lower values of the UI
EC_2019$Num_2019 <- paste(EC_2019$Num_2019,')',sep = '')   ##95% UI with brackets before and after
```

```
EC_2019$Num_2019 <- paste('(',EC_2019$Num_2019,sep = '')   ##95% UI with brackets before and after
EC_2019$Num_2019 <- paste(EC_2019$val,EC_2019$Num_2019,sep = '') ##Data and 95% of the UI are connected with the space bar


## 1990 ASR
ASR_1990 <- subset(EC,EC$year==1990 &
                     EC$age=='Age-standardized' &
                     EC$metric== 'Rate' &
                     EC$measure=='YLDs (Years Lived with Disability)'&
                     sex=='Female')
ASR_1990 <- subset(EC,EC$year==1990 &
                      EC$age=='Age-standardized' &
                      EC$metric== 'Rate' &
                      EC$measure=='YLDs (Years Lived with Disability)'&
                      sex=='Male')
ASR_1990 <- ASR_1990[,c(2,8,9,10)]   ### Take only the required variables: the region and the corresponding values
ASR_1990$val <- round(ASR_1990$val,2)   ###Rounding
ASR_1990$lower <- round(ASR_1990$lower,2)###Rounding
ASR_1990$upper <- round(ASR_1990$upper,2) ###Rounding
ASR_1990$ASR_1990 <- paste(ASR_1990$lower,ASR_1990$upper,sep = '-') ## Use - to connect 95% of the upper and lower values of the UI
ASR_1990$ASR_1990 <- paste(ASR_1990$ASR_1990,')',sep = '')   ##95% UI with brackets before and after
ASR_1990$ASR_1990 <- paste('(',ASR_1990$ASR_1990,sep = '')   ##95% UI with brackets before and after
ASR_1990$ASR_1990 <- paste(ASR_1990$val,ASR_1990$ASR_1990,sep = '') ##Data and 95% of the UI are connected with the space bar


## 2019 ASR
ASR_2019 <- subset(EC,EC$year==2019 &
                      EC$age=='Age-standardized' &
                      EC$metric== 'Rate' &
                      EC$measure=='YLDs (Years Lived with Disability)'&
                      sex=='Female')
ASR_2019 <- subset(EC,EC$year==2019 &
                      EC$age=='Age-standardized' &
                      EC$metric== 'Rate' &
                      EC$measure=='YLDs (Years Lived with Disability)'&
                      sex=='Male')
```

```
ASR_2019 <- ASR_2019[,c(2,8,9,10)]
ASR_2019$val <- round(ASR_2019$val,2)
ASR_2019$lower <- round(ASR_2019$lower,2)
ASR_2019$upper <- round(ASR_2019$upper,2)
ASR_2019$ASR_2019 <- paste(ASR_2019$lower,ASR_2019$upper,sep = '-')
ASR_2019$ASR_2019 <- paste(ASR_2019$ASR_2019,')',sep = '')
ASR_2019$ASR_2019 <- paste('(',ASR_2019$ASR_2019,sep = '')
ASR_2019$ASR_2019 <- paste(ASR_2019$val,ASR_2019$ASR_2019,sep = '')


##### Calculating EAPC
EAPC <- subset(EC, EC$age=='Age-standardized' &
                   EC$metric== 'Rate' &
                   EC$measure=='YLDs (Years Lived with Disability)'&
                   sex=='Female')
EAPC <- subset(EC, EC$age=='Age-standardized' &
                   EC$metric== 'Rate' &
                   EC$measure=='YLDs (Years Lived with Disability)'&
                   sex=='Male')

EAPC <- EAPC[,c(2,7,8)] ##Get the region, year and the corresponding value

country <- EC_1990$location
EAPC_cal <- data.frame(location=country,EAPC=rep(0,times=8),UCI=rep(0,times=8),LCI=rep(0,times=8))
for (i in 1:8){   ###A total of 8 regions, so 8 cycles
  country_cal <- as.character(EAPC_cal[i,1]) ### Take the corresponding areas in turn
  a <- subset(EAPC, EAPC$location==country_cal)   ##Take a subset of data from the corresponding region
  a$y <- log(a$val)   ##Calculate the y-value according to the EAPC calculation method
  mod_simp_reg<-lm(y~year,data=a) ##Do the linear regression equation according to the EAPC calculation method
  estimate <- (exp(summary(mod_simp_reg)[["coefficients"]][2,1])-1)*100 ##The EAPC is calculated by taking the beta value of the equation according to the EAPC calculation method
  low <- (exp(summary(mod_simp_reg)[["coefficients"]][2,1]-1.96*summary(mod_simp_reg)[["coefficients"]][2,2])-1)*100
  ### Calculate the upper limit of the 95% confidence interval for EAPC
  high <- (exp(summary(mod_simp_reg)[["coefficients"]][2,1]+1.96*summary(mod_simp_reg)[["coefficients"]][2,2])-1)*100
```

```
   ### Calculate the lower limit of the 95% confidence interval for EAPC
   EAPC_cal[i,2] <- estimate
   EAPC_cal[i,4] <- low
   EAPC_cal[i,3] <- high
}

EAPC_cal$EAPC <- round(EAPC_cal$EAPC,2)    ##Retain 2 decimal places
EAPC_cal$UCI <- round(EAPC_cal$UCI,2)
EAPC_cal$LCI <- round(EAPC_cal$LCI,2)
EAPC_cal$EAPC_CI <- paste(EAPC_cal$LCI,EAPC_cal$UCI,sep = '-')
EAPC_cal$EAPC_CI <- paste(EAPC_cal$EAPC_CI,')',sep = '')
EAPC_cal$EAPC_CI <- paste('(',EAPC_cal$EAPC_CI,sep = '')
EAPC_cal$EAPC_CI <-
paste(paste0(EAPC_cal$EAPC,'%'),EAPC_cal$EAPC_CI,sep = '')

###Change rate of YLDs
YLDs_change <- read.csv('region-change.csv')
EC_change <- subset(YLDs_change,
                    age=='All ages' &
                    metric== 'Rate' &
                    measure=='YLDs (Years Lived with Disability)'&
                    sex=='Female')
EC_change <- subset(YLDs_change,
                   age=='All ages' &
                     metric== 'Rate' &
                     measure=='YLDs (Years Lived with Disability)'&
                     sex=='Male')
EC_change <- EC_change[,c(2,9,10,11)]
EC_change$val <- round(EC_change$val,2)
EC_change$lower <- round(EC_change$lower,2)
EC_change$upper <- round(EC_change$upper,2)
EC_change$Num_change <- paste(EC_change$lower,EC_change$upper,sep = '-')
EC_change$Num_change <- paste(EC_change$Num_change,')',sep = '')
EC_change$Num_change <- paste('(',EC_change$Num_change,sep = '')
EC_change$Num_change <-
paste(paste0(EC_change$val,'%'),EC_change$Num_change,sep = '')


#整合数据

data <- flextable(YLDs, col_keys = names(YLDs))
data <- valign(data, valign = "center", part= "header")##Use the valign function to modify whether a table element is centered vertically, top-aligned or bottom-aligned
data <- align(data, align = "center", part= "all")##Use the align function to modify the alignment of table elements in the horizontal direction
```

```
data <- font(data, fontname = "Times New Roman", part = "all")##Use the font function to modify the font of elements in a table
data <- hline_top(data, border = fp_border(color="black", width = 1.5), part = "header") ##Use the hline_top function to modify the top line of the trilinear table, mainly in terms of color and line thickness
data <- hline_bottom(data, border = fp_border(color="black", width = 1.5), part = "body")##Use the hline_bottom function to make changes to the bottom line of the trilinear table
data <- hline(data, i=1, border = fp_border(color="black", width =1), part = "header")##Use the hline function to make changes to the line in the middle of the three-line table
data <- set_caption(data, "Table 1-2.YLDs of Tracheal, Bronchus, and Lung Cancer in 1990 and 2019 for both sexs in global and regions,with EAPC from 1990 and 2019.", autonum = 1, style = "Table Caption")##Use the function set_caption to set the title of the three-line table
data <- autofit(data) ##Automatic adjustment of the generated trilinear table using the autofit function

save_as_docx(data, path = "Table 1-2. YLDs of Tracheal, Bronchus, and Lung Cancer in 1990 and 2019 for both sex in global and regions, with EAPC from 1990 and 2019.docx") ##Use the save_as_docx function to generate the three-line table object exported to Word documents

write.csv(YLDs,'Results for YLDs nation.csv')##Exporting integrated data
```

2. The codes that were used for generating Figure 4 (A), and then save the image as a PDF and import it into Adobe Illustrator software to adjust the image size appropriately.

```
setwd('G:/GBD data/XM GBD mode/GBD_risk   factor code') ##Setting the work path
library(dplyr)
library(ggplot2)
library(ggsci)
### Risk factor for smoking
Risk <- read.csv('risk-YLDs-2019.csv',header = T)
order <- read.csv('order location.csv',header = F)
order$V1 <- rev(order$V1) ### Read the order of the different areas of the vertical coordinate
Risk_2019 <- subset(Risk, Risk$year==2019 &
                          Risk$sex=='Both' &
                          Risk$age=='All ages' &
                          Risk$rei=='Smoking' &
```

```
                              Risk$metric=='Percent')
Risk_2019$val <- round(Risk_2019$val*100,1)
Risk_2019$val2 <- paste0(Risk_2019$val,'%')
Risk_2019$location <- factor(Risk_2019$location,
                                              levels=order$V1,
                                              ordered=TRUE)    ###Let the vertical
coordinates - regions be displayed in our order
##Drawing
p1 <- ggplot(Risk_2019,aes(location,weight = val, fill = measure))+
   geom_bar(color = 'black',width = .7,position = 'dodge',
              size = .3)+
   scale_y_continuous(expand = c(0,0))+
   scale_fill_nejm() +
   theme_classic()+
   coord_flip() + facet_grid(.~rei) + theme_light() +
   geom_text(aes(label=val2, y=val+1.5), ### This value should be adjusted
according to the actual mapping results
               position=position_dodge(0.9), vjust=0,
               size = 2.5)
p1

## All risk factors
## year=2019
Risk_2019 <- subset(Risk, Risk$year==2019 &
                              Risk$sex=='Both' &
                              Risk$age=='All ages'&
                              Risk$metric=='Percent')
#Male
Risk_2019 <- subset(Risk, Risk$year==2019 &
                              Risk$sex=='Female' &
                              Risk$metric=='Percent'&
                           Risk$location=='Global')
write.csv(Risk_2019,'risk for YLDs (Female).csv')

#Female
Risk_2019 <- subset(Risk, Risk$year==2019 &
                              Risk$sex=='Male' &
                              Risk$location=='Global'&
                              Risk$metric=='Percent')
write.csv(Risk_2019,'risk for YLDs (Male).csv')

Risk_2019$val <- round(Risk_2019$val*100,1)
Risk_2019$val2 <- paste0(Risk_2019$val,'%')
Risk_2019$location <- factor(Risk_2019$location,
```

```
                                                  levels=order$V1,
                                                  ordered=TRUE)
Risk_2019$rei <- factor(Risk_2019$rei,
                                    levels= c('Smoking','Secondhand smoke',
                                              'Particulate matter pollution',
                                              'Occupational carcinogens',
                                              'High fasting plasma glucose',
                                              'Diet low in fruits'),
                                    ordered=TRUE)   ## Arrange the order in the order
you want
p1 <- ggplot(Risk_2019,aes(location,weight = val, fill = measure))+
  geom_bar(color = 'black',width = .4,position = 'dodge',
           size = .3)+
  scale_y_continuous(expand = c(0,0))+
  scale_fill_nejm() +
  theme_classic()+
  coord_flip() + facet_grid(.~rei) + theme_light() +
  geom_text(aes(label=val2, y=val+7.5),
            position=position_dodge(0.9), vjust=0,
            size = 2)
p1
```

3. **Figure 1** and **Figure 4** (B) is drawn by Excel 2019 without the use of R software. Therefore, the relevant code cannot be provided.
