# Supplementary material for: Global estimates of rehabilitation needs and disease burden in tracheal, bronchus, and lung cancer from 1990 to 2019 and projections to 2045 based on the global burden of disease study 2019
Source: Front Oncol. 2023 Jun 29;13:1152209. doi: 10.3389/fonc.2023.1152209 (PMC10344363; doi:10.3389/fonc.2023.1152209)
Supplement: Supplementary file 1 [file DataSheet_1.zip › Supplementary Material/Supplementary Material 6.pdf]

Table S2. Prevalence of Tracheal, Bronchus, and Lung Cancer in 1990 and 2019 for both sex in 204 countries, with EAPC from 1990 and 2019.

location	Num_1990	ASR_1990	Num_2019	ASR_2019	Num_change	EAPC_CI
Afghanistan	932(445-1609)	12.41(6.16-21.21)	1576(895-2505)	11.25(6.78-17.88)	-0.5%(-0.66--0.22)	-0.24%(-0.34--0.14)
Albania	680(635-727)	31.05(28.95-33.18)	1331(964-1798)	31.26(22.77-42.06)	1.38%(0.71-2.22)	0.01%(-0.21-0.24)
Algeria	1407(1124-1763)	11.21(9.06-14)	3359(2576-4327)	9.75(7.51-12.52)	0.44%(0.01-1.01)	-0.42%(-0.63--0.21)
American Samoa	6(5-7)	26.76(23.08-30.57)	13(11-15)	26.03(22.18-29.98)	0.75%(0.42-1.17)	0%(-0.04-0.03)
Andorra	32(25-44)	55.46(42.68-75.4)	87(66-110)	62.49(47.32-79.53)	0.76%(0.19-1.5)	0.41%(0.31-0.51)
Angola	598(387-853)	13.82(9.26-19.32)	1663(1278-2152)	13.64(10.76-17.45)	-0.05%(-0.36-0.48)	-0.1%(-0.23-0.03)
Antigua and Barbuda	5(5-6)	10.07(9.24-11.03)	11(9-13)	10.35(8.79-12.07)	0.44%(0.18-0.73)	0.17%(-0.07-0.42)
Argentina	10673(10314-11056)	32.62(31.53-33.82)	15089(11889-19082)	28.67(22.53-36.26)	0.04%(-0.19-0.31)	-0.65%(-0.73--0.56)
Armenia	1280(1214-1349)	41.85(39.76-44.04)	1490(1240-1771)	35.13(29.38-41.81)	0.32%(0.09-0.58)	-0.76%(-0.91--0.62)
Australia	10790(10323-11267)	55.19(52.8-57.61)	23935(18411-30510)	59.09(45.35-75.66)	0.52%(0.16-0.96)	0.38%(0.21-0.55)
Austria	4408(4185-4654)	40.25(38.22-42.56)	8918(7123-10869)	56.33(45.03-69.1)	0.76%(0.41-1.16)	1.37%(1.11-1.62)
Azerbaijan	1649(1501-1800)	29.23(26.73-31.81)	2746(2011-3522)	25.34(18.71-32.12)	0.19%(-0.12-0.55)	-0.16%(-0.62-0.31)

location	Num_1990	ASR_1990	Num_2019	ASR_2019	Num_change	EAPC_CI
Bahamas	28(26-32)	17.67(15.88-19.56)	66(53-83)	15.92(12.92-19.88)	0.57%(0.24-1.02)	-0.2%(-0.28--0.11)
Bahrain	58(49-68)	36.04(30.59-41.69)	155(117-205)	18.01(13.69-22.9)	-0.07%(-0.33-0.34)	-2.88%(-3.19--2.57)
Bangladesh	4298(3091-5596)	8.89(6.41-11.56)	9890(6398-15551)	7.4(4.82-11.58)	0.58%(0.02-1.29)	-0.61%(-0.73--0.49)
Barbados	27(25-29)	9.95(9.14-10.8)	53(43-64)	10.88(8.83-13.12)	0.67%(0.32-1.04)	0.15%(0.03-0.27)
Belarus	5111(4903-5317)	38.39(36.93-39.91)	4690(3580-6124)	29.73(22.75-39.08)	0.01%(-0.23-0.32)	-1.69%(-1.95--1.43)
Belgium	8894(8467-9361)	60.3(57.56-63.42)	12260(9448-15644)	59.89(45.8-76.82)	0.2%(-0.09-0.54)	0.16%(0.01-0.32)
Belize	10(9-11)	10.32(9.22-11.43)	42(36-49)	14.25(12.11-16.46)	0.96%(0.61-1.37)	0.88%(0.41-1.35)
Benin	186(154-220)	9.23(7.65-10.93)	483(359-640)	9.8(7.48-12.84)	0%(-0.26-0.35)	0.4%(0.32-0.47)
Bermuda	26(24-28)	41.38(38.36-44.75)	48(40-58)	37.9(31.6-46.02)	0.69%(0.38-1.11)	-0.06%(-0.19-0.06)
Bhutan	16(11-22)	5.9(4.1-8.36)	43(31-60)	7.58(5.44-10.45)	1.24%(0.55-2.3)	0.92%(0.87-0.98)
Bolivia (Plurinational State of)	405(272-528)	12.18(8.12-15.8)	1137(748-1569)	12.72(8.31-17.5)	0.5%(0.09-1.04)	0.01%(-0.09-0.11)
Bosnia and Herzegovina	1864(1748-1976)	40.87(38.54-43.15)	2754(2127-3521)	46.12(35.61-58.73)	1.03%(0.55-1.6)	0.39%(0.27-0.51)

location	Num_1990	ASR_1990	Num_2019	ASR_2019	Num_change	EAPC_CI
Botswana	110(84-140)	18(13.9-22.7)	326(224-441)	22.1(15.46-29.39)	0.65%(0.13-1.35)	0.25%(-0.07-0.56)
Brazil	16199(15749-16591)	17.33(16.8-17.76)	39012(36897-40901)	16.24(15.31-17.02)	0.65%(0.57-0.74)	-0.24%(-0.34--0.14)
Brunei Darussalam	46(37-54)	47.31(38.93-55.76)	159(139-180)	54.57(48.21-61.68)	1.05%(0.66-1.64)	0.82%(0.66-0.98)
Bulgaria	4148(3890-4415)	32.86(30.83-34.91)	5737(4490-7219)	45.03(34.97-57.09)	0.73%(0.36-1.19)	2.26%(1.8-2.72)
Burkina Faso	349(265-478)	7.74(5.93-10.54)	880(676-1183)	9.39(7.32-12.66)	0.06%(-0.18-0.38)	0.78%(0.6-0.97)
Burundi	185(138-242)	7.64(5.73-10.01)	320(222-447)	6.69(4.68-9.14)	-0.19%(-0.48-0.26)	-0.6%(-0.69--0.5)
Cabo Verde	24(22-27)	10.48(9.43-11.6)	71(57-86)	16.92(13.33-20.51)	0.85%(0.44-1.27)	1.18%(0.97-1.4)
Cambodia	1082(826-1387)	22.84(17.63-28.76)	2962(2290-3681)	23.96(18.86-29.67)	0.71%(0.22-1.32)	0.11%(0-0.22)
Cameroon	485(377-620)	10.47(8.17-13.33)	1587(1125-2202)	12.91(9.24-17.74)	0.17%(-0.2-0.67)	0.96%(0.86-1.06)
Canada	23853(22543-25339)	74.4(70.41-78.88)	55419(41888-71874)	81.97(61.93-106.42)	0.73%(0.31-1.25)	0.44%(0.2-0.68)
Central African Republic	183(92-304)	13.85(7.42-22.56)	301(148-535)	12.07(6.4-20.76)	-0.15%(-0.39-0.13)	-0.49%(-0.55--0.43)
Chad	217(157-287)	7.53(5.46-10.02)	618(439-881)	10.87(7.77-15.51)	0.05%(-0.23-0.39)	1.54%(1.44-1.65)

location	Num_1990	ASR_1990	Num_2019	ASR_2019	Num_change	EAPC_CI
Chile	1943(1861-2028)	19.03(18.24-19.86)	4682(3632-5919)	19.38(15.05-24.49)	0.76%(0.35-1.24)	0.38%(0.26-0.5)
China	279310(239824-320348)	31.05(26.74-35.48)	1137880(950548-1344733)	55.13(46.21-64.95)	2.39%(1.68-3.32)	2.24%(2.11-2.38)
Colombia	2859(2748-2964)	15.78(15.13-16.37)	7412(5749-9436)	14.08(10.9-17.87)	0.77%(0.36-1.25)	-0.64%(-0.77--0.5)
Comoros	16(9-21)	7.16(3.99-9.48)	35(25-47)	7.04(5.16-9.41)	0.4%(-0.08-1.64)	-0.26%(-0.41--0.1)
Congo	201(112-306)	17.32(10.05-25.92)	437(298-637)	15.49(10.98-22.01)	0.01%(-0.33-0.59)	-0.55%(-0.74--0.36)
Cook Islands	5(4-5)	34.83(29.54-40.7)	8(6-9)	30.57(25.89-36.21)	0.78%(0.39-1.34)	-0.52%(-0.59--0.45)
Costa Rica	210(197-222)	12.01(11.28-12.71)	596(459-762)	11.6(8.93-14.79)	0.83%(0.4-1.35)	-0.29%(-0.43--0.15)
Côte d'Ivoire	473(341-610)	11.12(8.15-14.32)	1308(949-1715)	11.99(8.9-15.3)	0.29%(-0.07-0.78)	0.21%(0.15-0.27)
Croatia	3854(3550-4195)	57.95(53.52-62.85)	5338(4123-6768)	65.31(50.42-83.33)	0.6%(0.22-1.04)	0.81%(0.58-1.03)
Cuba	3613(3464-3754)	35.13(33.64-36.5)	8223(6615-10108)	43.8(35.09-53.52)	1.17%(0.74-1.66)	0.97%(0.83-1.11)
Cyprus	169(145-195)	20.34(17.54-23.47)	738(627-857)	37.79(32.07-43.96)	1.59%(1.07-2.24)	2.98%(2.6-3.36)
Czechia	7540(7254-7881)	56.17(54.11-58.73)	9368(7574-11559)	45.93(36.89-56.75)	0.2%(-0.03-0.49)	-0.57%(-0.74--0.39)

location	Num_1990	ASR_1990	Num_2019	ASR_2019	Num_change	EAPC_CI
Democratic People's Republic of Korea	4855(3615-6496)	27.41(21.03-36.06)	9574(7289-12305)	29.05(22.14-37.09)	0.58%(0.18-1.07)	0.27%(0.21-0.33)
Democratic Republic of the Congo	2310(1255-5192)	13.51(7.59-29.99)	4472(2393-9049)	11.57(6.14-23.21)	-0.15%(-0.42-0.28)	-0.71%(-0.91--0.51)
Denmark	4794(4618-4975)	64.71(62.33-67.2)	7223(5576-9129)	66.41(50.97-83.61)	0.34%(0.03-0.69)	0.73%(0.38-1.09)
Djibouti	13(8-20)	8.58(5.55-12.93)	65(40-111)	10.31(6.6-17.08)	1.01%(0.35-2.01)	0.59%(0.54-0.64)
Dominica	11(10-13)	16.39(14.35-18.8)	16(13-19)	17.41(14-21.3)	0.5%(0.18-0.9)	0.36%(0.31-0.41)
Dominican Republic	433(381-492)	11.07(9.73-12.57)	1505(1070-2063)	15.91(11.4-21.77)	1.3%(0.57-2.26)	1.59%(1.46-1.73)
Ecuador	459(435-484)	8.31(7.87-8.78)	1507(1180-1948)	9.94(7.82-12.79)	0.87%(0.47-1.41)	0.7%(0.5-0.89)
Egypt	2173(1994-2385)	6.62(6.01-7.27)	6731(4752-9205)	9.46(6.65-12.78)	0.74%(0.19-1.4)	1.29%(1.17-1.41)
El Salvador	274(258-292)	8.93(8.39-9.51)	671(505-875)	11.44(8.57-14.91)	1.06%(0.52-1.72)	0.76%(0.65-0.87)
Equatorial Guinea	27(14-46)	12.34(6.57-20.76)	80(50-125)	15.8(10.22-24.28)	-0.1%(-0.57-0.71)	1.19%(1.04-1.34)
Eritrea	71(49-98)	6.27(4.49-8.53)	238(180-314)	8.11(6.27-10.38)	0.5%(0.04-1.23)	0.58%(0.39-0.77)
Estonia	884(850-921)	42.86(41.25-44.62)	806(626-1015)	33.31(25.74-42.23)	0.09%(-0.15-0.37)	-0.81%(-1.03--0.6)

location	Num_1990	ASR_1990	Num_2019	ASR_2019	Num_change	EAPC_CI
Eswatini	50(33-71)	15.97(10.75-22.77)	107(64-155)	17.39(10.59-24.68)	0.52%(0.04-1.27)	0.37%(0.2-0.55)
Ethiopia	1231(781-2178)	5.9(3.75-10.36)	2114(1477-2841)	5.16(3.59-6.98)	-0.18%(-0.46-0.51)	-0.6%(-0.73--0.47)
Fiji	38(31-47)	10.02(7.97-12.39)	75(59-96)	9.67(7.59-12.03)	0.64%(0.16-1.36)	-0.14%(-0.28-0.01)
Finland	3223(3064-3396)	46.25(43.99-48.68)	5056(3983-6452)	43(33.72-55.32)	0.42%(0.11-0.85)	0.03%(-0.08-0.13)
France	30790(29523-32157)	40.85(39.18-42.68)	67538(51577-87411)	59.39(45-76.7)	0.91%(0.47-1.47)	1.64%(1.43-1.84)
Gabon	109(63-166)	18.39(10.75-27.64)	212(139-308)	19.04(12.73-27.15)	0.1%(-0.24-0.61)	0.03%(-0.03-0.08)
Gambia	20(15-26)	5.66(4.29-7.19)	68(51-88)	7.06(5.34-9.13)	0.46%(-0.01-1.11)	0.6%(0.44-0.77)
Georgia	2285(2073-2510)	35.09(31.77-38.45)	1949(1614-2329)	34.83(28.85-41.54)	0.28%(0.04-0.57)	0.84%(0.3-1.39)
Germany	56509(53872-59489)	47.51(45.27-50.01)	102336(77684-133472)	61.97(46.93-80.98)	0.7%(0.29-1.22)	0.87%(0.58-1.17)
Ghana	537(431-661)	7.76(6.29-9.43)	1434(1137-1806)	8.39(6.77-10.55)	0.27%(-0.08-0.72)	0.03%(-0.12-0.19)
Greece	7719(7319-8174)	51.24(48.6-54.24)	12190(9412-15496)	60.46(46.18-77.22)	0.59%(0.23-1.01)	0.58%(0.43-0.73)
Greenland	33(29-37)	87.77(79.19-98.29)	61(50-72)	82.9(68.47-97.02)	0.84%(0.46-1.25)	-0.56%(-0.84--0.28)

location	Num_1990	ASR_1990	Num_2019	ASR_2019	Num_change	EAPC_CI
Grenada	10(9-11)	15.01(13.49-16.66)	18(16-20)	15.17(13.5-16.96)	0.45%(0.24-0.68)	0.23%(0.1-0.37)
Guam	30(26-34)	36.89(31.46-43.07)	63(52-75)	32.7(27.24-38.96)	0.7%(0.35-1.14)	-0.4%(-0.59--0.2)
Guatemala	355(313-404)	8.92(7.9-10.05)	908(703-1152)	7.87(6.11-9.97)	0.15%(-0.13-0.5)	-0.95%(-1.19--0.71)
Guinea	273(229-323)	8(6.73-9.42)	553(416-714)	9.71(7.39-12.52)	-0.01%(-0.27-0.34)	0.86%(0.81-0.92)
Guinea-Bissau	55(31-78)	12.88(7.56-18.24)	94(60-137)	12.37(7.94-17.79)	-0.09%(-0.36-0.28)	0.13%(-0.01-0.26)
Guyana	33(29-38)	8.33(7.17-9.5)	60(46-76)	8.92(6.91-11.32)	0.79%(0.32-1.38)	0.36%(0.26-0.47)
Haiti	462(273-740)	13.15(7.91-21.14)	809(497-1314)	10.98(6.78-17.91)	-0.1%(-0.38-0.35)	-0.49%(-0.66--0.33)
Honduras	301(239-360)	13.8(10.91-16.4)	1469(959-2123)	23.62(15.34-33.93)	1.34%(0.59-2.35)	1.98%(1.85-2.11)
Hungary	8063(7773-8355)	56.33(54.32-58.41)	11680(9517-14392)	66.56(54.22-82.49)	0.56%(0.27-0.9)	0.43%(0.12-0.74)
Iceland	142(130-156)	51.77(47.53-56.79)	341(292-397)	65.11(55.8-75.6)	0.77%(0.48-1.1)	0.82%(0.5-1.14)
India	30307(25734-35305)	6.3(5.34-7.34)	90058(73919-106987)	7.63(6.27-9.05)	0.83%(0.35-1.31)	0.53%(0.42-0.64)
Indonesia	17412(14951-19777)	16.5(14.18-18.74)	50234(36773-62098)	22.23(16.26-27.3)	1.06%(0.52-1.58)	0.97%(0.92-1.02)

location	Num_1990	ASR_1990	Num_2019	ASR_2019	Num_change	EAPC_CI
Iran (Islamic Republic of)	3080(2586-3688)	10.76(8.93-12.93)	9366(8689-10053)	12.53(11.58-13.47)	1.11%(0.67-1.64)	0.52%(0.32-0.72)
Iraq	1228(971-1525)	15.37(12.18-18.98)	4484(3428-5629)	18.76(14.44-23.11)	0.53%(0.1-1.09)	0.91%(0.61-1.22)
Ireland	1811(1720-1898)	44.63(42.42-46.82)	3777(2928-4844)	51.37(39.75-66)	0.53%(0.19-0.97)	0.71%(0.55-0.87)
Israel	1206(1148-1267)	25.53(24.23-26.83)	3480(2683-4484)	31.44(24.12-40.59)	0.54%(0.17-0.98)	0.5%(0.36-0.64)
Italy	41825(39995-43964)	48.46(46.43-50.8)	63160(51417-76261)	49.95(40.48-60.38)	0.42%(0.15-0.72)	0.18%(-0.01-0.37)
Jamaica	282(266-298)	16.52(15.59-17.49)	646(504-809)	21.8(17.07-27.35)	0.93%(0.49-1.43)	0.97%(0.46-1.48)
Japan	90558(86242-94625)	52.66(50.1-55.02)	253321(207167-301074)	74.49(62.1-88.37)	1.76%(1.29-2.26)	1.66%(1.37-1.95)
Jordan	179(147-221)	12.33(10.03-15.14)	1015(836-1233)	14.58(12.05-17.57)	0.83%(0.37-1.46)	1%(0.76-1.24)
Kazakhstan	6399(6075-6757)	46.69(44.3-49.25)	4337(3676-5019)	23.34(19.84-26.85)	-0.4%(-0.49--0.3)	-2.53%(-2.69--2.38)
Kenya	354(268-446)	4.25(3.21-5.33)	1204(986-1460)	5.31(4.41-6.4)	0.57%(0.25-1.03)	0.77%(0.66-0.89)
Kiribati	8(6-10)	19.07(15.31-23.2)	15(11-20)	18.86(13.98-24.6)	0.15%(-0.22-0.75)	-0.13%(-0.17--0.09)
Kuwait	78(69-86)	12.08(10.81-13.5)	259(216-310)	10.32(8.46-12.42)	0.32%(0.09-0.63)	-0.35%(-0.66--0.05)

location	Num_1990	ASR_1990	Num_2019	ASR_2019	Num_change	EAPC_CI
Kyrgyzstan	925(868-984)	29.08(27.34-30.9)	636(550-726)	12.91(11.21-14.63)	-0.53%(-0.6--0.46)	-2.98%(-3.46--2.49)
Lao People's Democratic Republic	545(371-770)	24.42(17.04-34.17)	1007(730-1330)	22(16.25-28.49)	0.07%(-0.3-0.55)	-0.61%(-0.72--0.51)
Latvia	1508(1442-1574)	41.92(40.12-43.71)	1259(1023-1549)	34.89(28.1-43.28)	0.16%(-0.07-0.42)	-0.6%(-0.89--0.31)
Lebanon	496(369-635)	20.61(15.41-26.17)	1577(1266-2051)	30.4(24.38-39.49)	1.01%(0.41-2.08)	2.04%(1.79-2.29)
Lesotho	124(95-177)	11.97(9.29-17.06)	237(158-337)	17.52(11.78-24.57)	0.66%(0.06-1.49)	1.56%(1.45-1.66)
Liberia	102(79-125)	8.93(7.02-10.99)	176(118-244)	8.55(5.86-11.78)	-0.29%(-0.52-0.01)	0.3%(0-0.6)
Libya	378(288-484)	19.61(15-25.08)	976(724-1248)	18.43(13.68-23.46)	0.62%(0.09-1.41)	-0.12%(-0.26-0.02)
Lithuania	1817(1745-1897)	39.92(38.38-41.68)	1673(1352-2043)	31.68(25.49-38.86)	0.21%(-0.02-0.49)	-0.73%(-0.93--0.54)
Luxembourg	276(260-296)	52.16(48.99-55.75)	482(392-590)	50.77(41.27-62.19)	0.07%(-0.13-0.33)	0.02%(-0.22-0.26)
Madagascar	352(288-428)	6.52(5.34-7.93)	710(513-947)	6.07(4.48-8.02)	-0.1%(-0.37-0.25)	-0.39%(-0.45--0.32)
Malawi	201(165-241)	5.07(4.2-6.03)	416(321-524)	5.59(4.39-6.97)	0.07%(-0.19-0.37)	0.32%(0.19-0.45)
Malaysia	1518(1333-1750)	16.26(14.27-18.73)	5544(4258-7066)	20.2(15.66-25.69)	1.06%(0.52-1.79)	0.67%(0.41-0.93)

location	Num_1990	ASR_1990	Num_2019	ASR_2019	Num_change	EAPC_CI
Maldives	11(8-15)	12.62(8.79-16.87)	29(24-35)	9.72(7.95-11.65)	0.15%(-0.24-0.83)	-1.39%(-1.6--1.17)
Mali	252(211-296)	5.84(4.91-6.8)	600(451-796)	6.84(5.22-8.92)	-0.06%(-0.32-0.29)	0.61%(0.55-0.67)
Malta	127(118-138)	29.39(27.22-31.87)	265(223-314)	30.63(25.7-36.52)	0.76%(0.46-1.14)	0.34%(0.24-0.44)
Marshall Islands	5(3-7)	25.8(15.67-39.42)	11(6-16)	28.72(17.81-42.33)	0.88%(0.36-1.57)	0.57%(0.46-0.68)
Mauritania	104(82-128)	10.09(7.9-12.32)	211(142-301)	10.05(6.93-14.04)	0.04%(-0.32-0.65)	0.27%(0.08-0.47)
Mauritius	109(102-116)	14.09(13.25-15.05)	221(180-270)	12.39(10.13-15.04)	0.75%(0.39-1.16)	-0.59%(-0.68--0.49)
Mexico	5996(5840-6104)	13.79(13.33-14.07)	11875(10225-13705)	10.12(8.73-11.67)	0.36%(0.17-0.56)	-1.58%(-1.76--1.41)
Micronesia (Federated States of)	13(9-20)	26.6(18.72-38.72)	24(14-36)	30.68(18.38-45.54)	0.8%(0.16-1.64)	0.48%(0.45-0.51)
Monaco	37(29-45)	63.19(49.98-76.91)	97(78-118)	119(94.66-147.2)	1.11%(0.57-1.81)	2.65%(2.18-3.11)
Mongolia	405(329-487)	38.07(31.09-45.61)	691(532-911)	29.55(23.23-38.17)	0.09%(-0.21-0.51)	-1.55%(-1.8--1.29)
Montenegro	375(326-423)	57.57(50.2-64.83)	692(568-840)	70.6(57.82-85.83)	0.86%(0.46-1.37)	0.91%(0.79-1.03)
Morocco	2150(1634-2663)	14.73(11.23-18.12)	5602(4046-7309)	16.63(11.96-21.41)	0.83%(0.28-1.53)	0.3%(0.16-0.43)

location	Num_1990	ASR_1990	Num_2019	ASR_2019	Num_change	EAPC_CI
Mozambique	297(242-354)	4.91(4.04-5.78)	856(631-1151)	7.66(5.72-10.13)	0.27%(-0.11-0.73)	1.93%(1.8-2.06)
Myanmar	5874(4013-9130)	23.69(16.35-36.21)	10552(7740-14581)	21.88(16.11-29.98)	0.35%(-0.06-1.01)	-0.4%(-0.45--0.36)
Namibia	44(36-54)	5.96(4.89-7.29)	107(82-139)	7.49(5.84-9.57)	0.41%(0.06-0.92)	0.8%(0.73-0.87)
Nauru	2(1-2)	38.27(25.67-53.29)	2(1-2)	37.33(23.86-49.8)	0.08%(-0.21-0.46)	-0.22%(-0.29--0.14)
Nepal	681(461-962)	6.83(4.56-9.73)	1764(1267-2294)	7.71(5.53-9.98)	0.66%(0.17-1.35)	0.34%(0.15-0.52)
Netherlands	11957(11381-12560)	62.02(59.04-65.07)	23169(17992-29380)	71.87(55.63-91.28)	0.69%(0.29-1.14)	0.79%(0.58-1.01)
New Zealand	1914(1799-2028)	49.08(46.28-51.9)	4246(3482-5043)	55.33(45.59-65.68)	0.69%(0.38-1.05)	0.47%(0.36-0.58)
Nicaragua	117(104-131)	7.4(6.5-8.48)	404(326-494)	9.14(7.4-11.06)	1.07%(0.61-1.61)	0.61%(0.5-0.72)
Niger	229(157-319)	7.86(5.37-10.89)	656(409-958)	8.33(5.3-12.02)	-0.01%(-0.28-0.36)	0.44%(0.29-0.6)
Nigeria	2591(1872-3496)	5.83(4.27-7.79)	5794(4462-7517)	6.85(5.34-8.75)	-0.06%(-0.35-0.35)	0.86%(0.74-0.99)
Niue	1(0-1)	26.85(22.1-32.95)	1(1-1)	30.71(24.29-38.24)	0.63%(0.22-1.15)	0.48%(0.43-0.54)
North Macedonia	664(609-722)	32.81(30.12-35.61)	1598(1208-2059)	48.11(36.56-61.85)	1.25%(0.7-1.95)	1.38%(1.17-1.6)

location	Num_1990	ASR_1990	Num_2019	ASR_2019	Num_change	EAPC_CI
Northern Mariana Islands	11(9-13)	55.91(48.1-65.89)	26(22-30)	46.99(40.48-53.3)	1.59%(1.06-2.22)	-0.79%(-0.89--0.69)
Norway	2357(2227-2477)	38.09(36.15-39.97)	5377(4455-6382)	58.13(48.21-68.66)	0.81%(0.51-1.14)	1.94%(1.52-2.36)
Oman	62(45-81)	8.89(6.48-11.48)	169(132-224)	9.74(7.92-12.01)	0.15%(-0.21-0.72)	0.56%(0.28-0.83)
Pakistan	7977(6719-9263)	13.64(11.45-15.89)	19632(14826-26083)	16.44(12.48-21.7)	0.24%(-0.11-0.76)	0.54%(0.3-0.77)
Palau	4(3-5)	41.49(32.88-52.95)	10(8-13)	45.43(36.2-56.64)	1.07%(0.49-1.77)	0.28%(0.23-0.32)
Palestine	176(130-233)	19.45(14.51-25.84)	568(481-667)	22.43(18.96-26.21)	0.35%(-0.06-0.89)	0.59%(0.35-0.82)
Panama	225(211-239)	14.93(13.95-15.87)	511(391-654)	12.37(9.5-15.85)	0.3%(-0.01-0.68)	-0.98%(-1.14--0.81)
Papua New Guinea	347(235-536)	17.32(11.83-27.04)	1036(714-1580)	20.39(14.46-30.87)	0.24%(-0.1-0.77)	0.59%(0.56-0.61)
Paraguay	240(212-274)	10.56(9.34-12.06)	909(682-1174)	16.19(12.19-20.83)	1.21%(0.61-1.96)	1.29%(1.03-1.55)
Peru	1823(1557-2129)	14.7(12.58-17.17)	3527(2640-4681)	10.99(8.23-14.58)	0.24%(-0.11-0.74)	-1.03%(-1.37--0.69)
Philippines	7213(6339-8208)	22.75(20.04-25.73)	14616(11581-18202)	17.65(14.07-21.88)	0.14%(-0.13-0.54)	-1.51%(-1.77--1.25)
Poland	20546(20210-20883)	46.56(45.79-47.32)	30293(25281-36146)	44.54(37.08-53.18)	0.46%(0.22-0.75)	-0.3%(-0.47--0.13)

location	Num_1990	ASR_1990	Num_2019	ASR_2019	Num_change	EAPC_CI
Portugal	2807(2706-2909)	20.48(19.77-21.22)	5227(4021-6686)	25.58(19.5-32.89)	0.77%(0.35-1.28)	0.96%(0.67-1.24)
Puerto Rico	649(617-683)	17.92(17.06-18.83)	1084(839-1386)	16.26(12.54-20.94)	0.71%(0.33-1.22)	-0.35%(-0.46--0.25)
Qatar	18(14-24)	16.31(12.37-21.29)	153(107-213)	17.54(13.23-22.69)	0.3%(-0.2-1.13)	0.54%(0.23-0.85)
Republic of Korea	9381(8911-9929)	28.66(27.24-30.46)	68345(55468-83426)	75.66(61.36-92.07)	5.05%(3.9-6.49)	3.03%(2.64-3.43)
Republic of Moldova	1582(1510-1652)	33.53(32.08-35.02)	1218(1047-1407)	21.03(18.03-24.26)	-0.07%(-0.21-0.07)	-0.94%(-1.38--0.49)
Romania	8882(8534-9227)	30.45(29.29-31.58)	13734(11257-16703)	41.06(33.48-49.93)	0.88%(0.54-1.29)	0.88%(0.74-1.03)
Russian Federation	72386(69849-73811)	38.26(36.87-39.04)	74012(63149-86679)	31.52(26.9-36.93)	0.05%(-0.1-0.23)	-0.87%(-1.15--0.6)
Rwanda	252(198-317)	8.28(6.56-10.29)	482(332-710)	7.76(5.36-11.3)	0.08%(-0.3-0.82)	-0.55%(-0.77--0.33)
Saint Kitts and Nevis	5(4-5)	13.12(11.87-14.37)	9(7-11)	12.53(10.29-15.02)	0.32%(0.05-0.63)	-0.37%(-0.56--0.18)
Saint Lucia	12(11-13)	13.75(12.65-14.91)	29(24-35)	13.28(11.18-15.76)	0.9%(0.57-1.3)	-0.25%(-0.46--0.05)
Saint Vincent and the Grenadines	7(6-7)	9.49(8.65-10.41)	15(13-17)	10.66(9.24-12.31)	1.1%(0.79-1.49)	0.33%(0.21-0.45)
Samoa	10(8-12)	11.13(9.03-13.35)	17(13-22)	11.32(8.77-14.41)	0.31%(-0.03-0.78)	0.03%(0-0.06)

location	Num_1990	ASR_1990	Num_2019	ASR_2019	Num_change	EAPC_CI
San Marino	15(13-17)	45.53(39.46-52.36)	33(24-43)	57.2(42.27-75.82)	0.57%(0.14-1.15)	1.12%(1.01-1.23)
Sao Tome and Principe	9(7-10)	12.94(10.26-15.53)	20(15-25)	18.01(14.01-22.56)	0.34%(0-0.81)	1.16%(1.09-1.22)
Saudi Arabia	434(320-566)	7.21(5.39-9.3)	1848(1411-2305)	9.01(7.13-10.91)	0.91%(0.29-1.9)	0.71%(0.61-0.81)
Senegal	336(248-432)	10.12(7.57-13)	863(639-1141)	11.37(8.58-14.92)	0.29%(-0.06-0.8)	0.72%(0.47-0.96)
Serbia	5392(4515-5937)	43.62(36.72-47.74)	9490(7405-12061)	63.36(49.19-80.68)	0.89%(0.44-1.5)	1.55%(1.37-1.72)
Seychelles	10(9-11)	18.09(15.73-20.22)	20(18-24)	17.92(15.66-20.75)	0.42%(0.17-0.7)	-0.32%(-0.46--0.19)
Sierra Leone	174(132-218)	9(6.9-11.24)	357(251-482)	9.79(6.95-13.19)	-0.1%(-0.35-0.22)	0.62%(0.4-0.83)
Singapore	1228(1155-1302)	54.94(51.6-58.3)	4818(3741-6168)	61.39(48.02-78.24)	1.11%(0.65-1.7)	0.52%(0.39-0.66)
Slovakia	3911(3660-4200)	65.77(61.68-70.65)	5222(3981-6781)	56.7(43.32-73.57)	0.3%(-0.03-0.73)	-0.35%(-0.73-0.03)
Slovenia	1070(818-1391)	43.48(33.3-56.32)	1981(1511-2609)	51.07(38.75-67.39)	0.76%(0.17-1.53)	0.7%(0.45-0.95)
Solomon Islands	44(23-74)	28.59(15.97-47.1)	112(57-185)	32.53(17.81-51.95)	0.32%(-0.07-0.83)	0.42%(0.34-0.49)
Somalia	170(111-266)	6.3(4.23-9.67)	378(212-635)	5.32(3.01-8.76)	-0.22%(-0.5-0.12)	-0.51%(-0.57--0.46)

location	Num_1990	ASR_1990	Num_2019	ASR_2019	Num_change	EAPC_CI
South Africa	4912(4100-6451)	22.4(18.6-29.78)	8878(7900-10194)	19.17(17.1-21.95)	0.2%(-0.02-0.41)	-0.79%(-1.08--0.51)
South Sudan	225(148-343)	9.22(6.11-14.02)	317(205-458)	8.16(5.35-11.64)	-0.11%(-0.38-0.3)	-0.45%(-0.52--0.39)
Spain	24724(23473-26060)	46.93(44.62-49.39)	49700(37825-64080)	59.35(44.88-77.64)	0.69%(0.28-1.21)	0.88%(0.72-1.03)
Sri Lanka	903(790-1016)	7.77(6.8-8.72)	2834(2039-3892)	10.73(7.78-14.6)	1.47%(0.74-2.42)	1.77%(1.57-1.97)
Sudan	713(406-1297)	7.36(4.25-13.48)	1589(1011-2462)	8.03(5.16-12.46)	0.1%(-0.29-0.84)	0.33%(0.28-0.38)
Suriname	35(32-39)	13.17(11.95-14.42)	97(79-117)	15.61(12.84-18.89)	0.83%(0.47-1.27)	0.61%(0.37-0.85)
Sweden	3122(2997-3240)	23.18(22.26-24.11)	4990(4224-5805)	25.73(21.72-30.01)	0.34%(0.14-0.57)	0.4%(0.21-0.58)
Switzerland	4983(4733-5265)	52.04(49.4-55.13)	6642(5129-8518)	42.12(32.44-54.4)	0.04%(-0.2-0.35)	-0.39%(-0.59--0.19)
Syrian Arab Republic	587(453-738)	10.05(7.71-12.64)	1509(1100-2011)	11.37(8.39-15.01)	1.29%(0.55-2.42)	0.46%(0.28-0.64)
Taiwan (Province of China)	4535(4393-4689)	26.77(25.9-27.62)	16026(12395-20728)	40.73(31.55-52.71)	2.05%(1.36-2.94)	1.39%(0.83-1.94)
Tajikistan	645(588-717)	21.2(19.4-23.48)	665(532-838)	11.93(9.67-14.81)	-0.42%(-0.54--0.26)	-1.38%(-1.86--0.9)
Thailand	11224(9955-12562)	29.11(25.9-32.5)	24361(18211-32138)	23.76(17.79-31.24)	0.76%(0.28-1.39)	-1.21%(-1.39--1.04)

location	Num_1990	ASR_1990	Num_2019	ASR_2019	Num_change	EAPC_CI
Timor-Leste	48(36-64)	15.62(11.97-20.65)	160(113-209)	18.82(13.4-24.66)	0.97%(0.32-1.81)	0.76%(0.52-0.99)
Togo	124(100-155)	9.64(7.78-12.03)	404(291-542)	10.6(7.84-14.06)	0.51%(0.08-1.08)	0.4%(0.35-0.46)
Tokelau	0(0-0)	20.42(15.98-26.03)	0(0-0)	23.86(18.49-30.98)	0.39%(0.02-0.87)	0.59%(0.56-0.63)
Tonga	14(11-18)	25.15(19.81-31.44)	21(17-26)	26.8(21.71-32.72)	0.4%(0.03-0.88)	0.16%(0.01-0.32)
Trinidad and Tobago	103(97-110)	12(11.23-12.79)	214(160-282)	11.41(8.53-15.01)	0.8%(0.34-1.39)	-0.19%(-0.3--0.07)
Tunisia	971(777-1172)	18.54(14.88-22.33)	2592(1812-3593)	19.87(14.07-27.4)	0.95%(0.29-1.9)	0.01%(-0.09-0.1)
Turkey	14712(11389-18406)	38.27(29.91-47.54)	31740(25031-39550)	34.95(27.58-43.51)	0.58%(0.12-1.24)	0.36%(-0.38-1.11)
Turkmenistan	407(387-427)	18.68(17.76-19.6)	478(376-606)	10.82(8.57-13.61)	-0.14%(-0.33-0.09)	-2.21%(-2.82--1.6)
Tuvalu	2(1-2)	22.98(17.61-33.49)	3(2-4)	25.04(18.42-34.17)	0.25%(-0.1-0.74)	0.23%(0.2-0.26)
Uganda	370(297-446)	5.54(4.5-6.63)	1035(817-1263)	7.02(5.61-8.41)	0.18%(-0.11-0.57)	0.69%(0.63-0.76)
Ukraine	40787(38780-42943)	56.47(53.75-59.43)	30170(24475-36910)	41.65(33.82-50.95)	-0.12%(-0.29-0.09)	-1.82%(-2.24--1.4)
United Arab Emirates	67(50-87)	17.08(12.77-21.97)	627(454-835)	16.87(12.24-23.29)	0.91%(0.22-1.98)	-0.12%(-0.49-0.25)

location	Num_1990	ASR_1990	Num_2019	ASR_2019	Num_change	EAPC_CI
United Kingdom	54867(53284-56013)	61.95(60.27-63.18)	82806(69271-97595)	67.09(55.84-79.44)	0.29%(0.08-0.52)	0.47%(0.37-0.58)
United Republic of Tanzania	879(642-1192)	7.73(5.69-10.41)	2154(1527-3125)	8.59(6.18-12.39)	0.12%(-0.15-0.43)	0.33%(0.27-0.4)
United States of America	282848(275206-289445)	92.42(90.07-94.46)	444083(384699-516048)	80.19(69.4-93.32)	0.21%(0.05-0.41)	-0.81%(-1.01--0.6)
United States Virgin Islands	16(13-20)	18.18(15.2-21.72)	49(40-57)	26.55(21.73-31.18)	2.08%(1.42-2.96)	1.79%(1.52-2.06)
Uruguay	1774(1689-1863)	46.97(44.8-49.41)	1937(1500-2435)	39.46(30.39-49.94)	0%(-0.24-0.28)	-0.58%(-0.7--0.46)
Uzbekistan	2462(2350-2575)	19.9(19.02-20.79)	3247(2682-3914)	13.26(11.04-15.75)	-0.18%(-0.32--0.01)	-1.96%(-2.27--1.64)
Vanuatu	13(8-22)	19.49(12.32-31.01)	42(28-63)	23.36(15.51-34.99)	0.62%(0.13-1.63)	0.52%(0.37-0.66)
Venezuela (Bolivarian Republic of)	1964(1880-2047)	19.4(18.55-20.23)	6673(4864-8706)	22.34(16.41-29.01)	1.28%(0.67-1.99)	0.59%(0.36-0.82)
Viet Nam	8943(6997-10976)	21.38(16.79-26.26)	28987(22215-37389)	28.89(22.25-36.69)	1.29%(0.64-2.16)	1.09%(1.05-1.13)
Yemen	481(300-768)	9.12(5.85-14.37)	1345(909-2008)	9.57(6.56-14.22)	0.22%(-0.17-0.91)	0.35%(0.28-0.42)
Zambia	275(220-348)	9.27(7.44-11.68)	725(516-959)	10.31(7.43-13.4)	0.15%(-0.19-0.56)	0.2%(0.14-0.25)
Zimbabwe	563(493-637)	13.06(11.49-14.77)	1059(806-1300)	14.11(10.91-17.15)	0.3%(-0.02-0.66)	0.32%(0.21-0.43)

Table S3. YLDs of Tracheal, Bronchus, and Lung Cancer in 1990 and 2019 for both sex in 204 countries, with EAPC from 1990 and 2019.

location	Num_1990	ASR_1990	Num_2019	ASR_2019	Num_change	EAPC_CI
Afghanistan	217(93-421)	2.97(1.33-5.66)	346(177-584)	2.62(1.44-4.41)	-0.52%(-0.69--0.23)	-0.33%(-0.42--0.25)
Albania	142(100-188)	6.67(4.71-8.75)	269(166-405)	6.26(3.87-9.45)	1.31%(0.6-2.17)	-0.24%(-0.45--0.02)
Algeria	322(203-463)	2.67(1.71-3.78)	750(487-1095)	2.25(1.45-3.25)	0.41%(-0.05-1.06)	-0.51%(-0.71--0.31)
American Samoa	1(1-2)	6.13(4.2-8.08)	3(2-4)	5.75(4.1-7.71)	0.73%(0.33-1.23)	-0.12%(-0.16--0.08)
Andorra	6(4-9)	10.53(7.02-15.43)	14(9-19)	9.9(6.43-13.93)	0.5%(0-1.16)	-0.24%(-0.28--0.21)
Angola	135(76-215)	3.28(1.91-5.15)	367(238-542)	3.17(2.09-4.59)	-0.07%(-0.4-0.5)	-0.16%(-0.29--0.04)
Antigua and Barbuda	1(1-2)	2.29(1.57-3.11)	2(2-3)	2.21(1.47-2.98)	0.3%(-0.01-0.7)	-0.04%(-0.27-0.19)
Argentina	2308(1616-2983)	7.08(4.94-9.14)	3166(2056-4556)	5.96(3.84-8.59)	0.01%(-0.25-0.34)	-0.77%(-0.85--0.69)
Armenia	258(181-342)	8.63(6.09-11.33)	307(214-416)	7.26(5.01-9.87)	0.35%(0.04-0.7)	-0.72%(-0.87--0.56)
Australia	1794(1290-2295)	9.12(6.54-11.71)	3289(2235-4679)	7.98(5.41-11.39)	0.26%(-0.05-0.63)	-0.42%(-0.52--0.31)
Austria	818(586-1055)	7.27(5.23-9.45)	1296(875-1789)	7.92(5.33-10.86)	0.38%(0.06-0.77)	0.48%(0.32-0.64)
Azerbaijan	342(234-455)	6.22(4.29-8.32)	563(346-817)	5.42(3.39-7.73)	0.17%(-0.2-0.64)	-0.14%(-0.57-0.29)

location	Num_1990	ASR_1990	Num_2019	ASR_2019	Num_change	EAPC_CI
Bahamas	6(4-8)	3.88(2.69-5.17)	14(9-19)	3.39(2.27-4.71)	0.52%(0.12-1.05)	-0.33%(-0.42--0.25)
Bahrain	13(9-17)	8.61(5.9-11.32)	33(21-49)	4.15(2.76-5.86)	-0.12%(-0.4-0.3)	-2.99%(-3.3--2.69)
Bangladesh	998(596-1493)	2.12(1.25-3.2)	2281(1252-4049)	1.74(0.96-3.07)	0.56%(-0.06-1.42)	-0.65%(-0.77--0.53)
Barbados	6(4-8)	2.25(1.54-3.05)	11(7-16)	2.29(1.51-3.15)	0.52%(0.13-0.96)	-0.14%(-0.25--0.02)
Belarus	1040(728-1350)	7.83(5.5-10.15)	890(575-1271)	5.61(3.61-8.05)	-0.06%(-0.31-0.28)	-1.96%(-2.22--1.7)
Belgium	1782(1285-2318)	11.88(8.55-15.44)	2055(1357-2935)	9.7(6.38-13.98)	0.01%(-0.23-0.32)	-0.58%(-0.75--0.41)
Belize	2(2-3)	2.4(1.65-3.28)	9(6-12)	3.11(2.08-4.27)	0.8%(0.37-1.31)	0.64%(0.18-1.09)
Benin	44(30-61)	2.26(1.49-3.09)	112(72-165)	2.37(1.52-3.43)	-0.03%(-0.31-0.38)	0.35%(0.28-0.43)
Bermuda	6(4-7)	8.83(6.16-11.55)	8(6-11)	6.52(4.48-8.95)	0.4%(0.11-0.81)	-0.86%(-0.98--0.74)
Bhutan	4(2-6)	1.43(0.82-2.22)	10(6-15)	1.78(1.11-2.7)	1.23%(0.47-2.37)	0.81%(0.77-0.86)
Bolivia (Plurinational State of)	93(51-139)	2.88(1.58-4.32)	261(152-401)	2.99(1.73-4.62)	0.5%(0.02-1.13)	0%(-0.1-0.1)
Bosnia and Herzegovina	381(273-499)	8.6(6.16-11.22)	548(350-786)	9.13(5.82-12.99)	0.98%(0.44-1.62)	0.14%(0.03-0.25)

location	Num_1990	ASR_1990	Num_2019	ASR_2019	Num_change	EAPC_CI
Botswana	24(16-35)	4.06(2.66-5.89)	68(42-104)	4.81(2.95-7.2)	0.58%(0.01-1.37)	0.15%(-0.16-0.46)
Brazil	3524(2494-4482)	3.87(2.75-4.91)	8344(5981-10706)	3.51(2.51-4.5)	0.63%(0.51-0.75)	-0.36%(-0.45--0.27)
Brunei Darussalam	9(6-12)	10.02(6.81-13.36)	27(19-36)	10.25(7.29-13.39)	0.79%(0.38-1.38)	0.47%(0.3-0.64)
Bulgaria	836(592-1102)	6.61(4.69-8.71)	1117(743-1572)	8.55(5.57-12.08)	0.67%(0.25-1.2)	2.05%(1.59-2.52)
Burkina Faso	83(51-122)	1.91(1.18-2.81)	203(128-302)	2.26(1.44-3.35)	0.03%(-0.26-0.43)	0.7%(0.53-0.87)
Burundi	45(28-65)	1.91(1.21-2.76)	75(45-117)	1.65(0.99-2.52)	-0.22%(-0.52-0.23)	-0.65%(-0.75--0.55)
Cabo Verde	6(4-8)	2.47(1.74-3.29)	16(10-22)	3.84(2.48-5.32)	0.74%(0.29-1.32)	1.05%(0.83-1.27)
Cambodia	241(156-342)	5.27(3.45-7.46)	650(420-907)	5.43(3.56-7.61)	0.69%(0.17-1.37)	0.05%(-0.05-0.14)
Cameroon	111(70-159)	2.49(1.6-3.55)	362(217-558)	3.08(1.88-4.61)	0.16%(-0.24-0.7)	0.91%(0.82-1.01)
Canada	4050(2932-5242)	12.55(9.07-16.22)	7744(5129-10899)	11.31(7.43-16.03)	0.43%(0.08-0.88)	-0.37%(-0.54--0.19)
Central African Republic	41(19-76)	3.26(1.6-5.83)	67(30-130)	2.85(1.38-5.26)	-0.15%(-0.43-0.21)	-0.47%(-0.54--0.4)
Chad	52(31-80)	1.85(1.1-2.83)	143(86-223)	2.62(1.57-4.04)	0.01%(-0.3-0.43)	1.43%(1.34-1.53)

location	Num_1990	ASR_1990	Num_2019	ASR_2019	Num_change	EAPC_CI
Chile	424(299-556)	4.21(2.97-5.51)	930(604-1334)	3.85(2.5-5.53)	0.6%(0.18-1.14)	-0.03%(-0.14-0.07)
China	59316(41688-77779)	6.81(4.78-8.9)	199352(138983-264036)	9.84(6.88-12.99)	1.8%(1.21-2.58)	1.48%(1.33-1.62)
Colombia	620(440-830)	3.52(2.48-4.71)	1506(962-2199)	2.86(1.82-4.15)	0.65%(0.2-1.19)	-0.96%(-1.08--0.83)
Comoros	4(2-6)	1.77(0.97-2.68)	8(5-13)	1.71(1.07-2.58)	0.37%(-0.11-1.54)	-0.31%(-0.45--0.16)
Congo	45(21-76)	4.01(2.01-6.68)	97(57-154)	3.59(2.17-5.62)	0%(-0.37-0.67)	-0.54%(-0.72--0.36)
Cook Islands	1(1-1)	7.88(5.36-10.7)	2(1-2)	6.43(4.4-8.69)	0.7%(0.27-1.26)	-0.77%(-0.84--0.71)
Costa Rica	45(31-60)	2.6(1.81-3.49)	119(75-173)	2.33(1.47-3.39)	0.71%(0.22-1.27)	-0.59%(-0.72--0.45)
Côte d'Ivoire	107(64-158)	2.64(1.61-3.88)	297(192-434)	2.87(1.85-4.17)	0.3%(-0.11-0.9)	0.22%(0.16-0.29)
Croatia	700(488-925)	10.58(7.39-13.98)	836(552-1174)	10.03(6.57-14.15)	0.38%(0.03-0.86)	0.17%(-0.06-0.39)
Cuba	774(538-998)	7.52(5.22-9.71)	1563(1063-2173)	8.27(5.61-11.55)	0.93%(0.49-1.44)	0.53%(0.41-0.65)
Cyprus	36(25-48)	4.36(3.06-5.74)	124(88-168)	6.32(4.5-8.52)	1.05%(0.58-1.68)	1.89%(1.59-2.19)
Czechia	1506(1080-1950)	11.13(7.92-14.4)	1604(1073-2221)	7.74(5.19-10.73)	0.03%(-0.2-0.32)	-1.12%(-1.28--0.96)

location	Num_1990	ASR_1990	Num_2019	ASR_2019	Num_change	EAPC_CI
Democratic People's Republic of Korea	1044(659-1536)	6.11(3.96-8.84)	2084(1353-2985)	6.38(4.17-9.12)	0.6%(0.14-1.2)	0.22%(0.17-0.27)
Democratic Republic of the Congo	521(244-1244)	3.19(1.49-7.44)	989(459-2131)	2.67(1.23-5.58)	-0.16%(-0.45-0.33)	-0.73%(-0.91--0.55)
Denmark	933(675-1194)	12.25(8.86-15.64)	1180(796-1660)	10.5(6.99-14.82)	0.12%(-0.14-0.44)	-0.17%(-0.41-0.07)
Djibouti	3(2-5)	2.09(1.19-3.39)	15(7-26)	2.45(1.27-4.28)	0.99%(0.23-2.13)	0.5%(0.46-0.55)
Dominica	3(2-3)	3.64(2.5-4.87)	3(2-5)	3.83(2.53-5.42)	0.46%(0.09-0.98)	0.31%(0.25-0.37)
Dominican Republic	97(66-132)	2.56(1.75-3.5)	327(202-494)	3.51(2.19-5.3)	1.23%(0.46-2.32)	1.45%(1.31-1.58)
Ecuador	106(72-143)	1.99(1.35-2.69)	339(221-482)	2.28(1.49-3.24)	0.82%(0.35-1.42)	0.57%(0.37-0.77)
Egypt	492(333-656)	1.57(1.05-2.11)	1475(859-2284)	2.16(1.25-3.31)	0.69%(0.09-1.47)	1.18%(1.05-1.31)
El Salvador	61(42-82)	2.04(1.4-2.75)	144(91-209)	2.44(1.53-3.56)	0.98%(0.39-1.72)	0.52%(0.41-0.63)
Equatorial Guinea	6(3-11)	2.91(1.4-5.27)	18(10-29)	3.65(2.04-5.78)	-0.12%(-0.57-0.71)	1.08%(0.93-1.22)
Eritrea	17(9-26)	1.57(0.91-2.4)	54(35-79)	1.96(1.26-2.8)	0.45%(-0.04-1.21)	0.47%(0.26-0.68)
Estonia	186(135-242)	9(6.59-11.71)	163(108-233)	6.54(4.33-9.39)	0.05%(-0.2-0.36)	-1.13%(-1.33--0.93)

location	Num_1990	ASR_1990	Num_2019	ASR_2019	Num_change	EAPC_CI
Eswatini	11(6-17)	3.66(2.14-5.59)	23(12-37)	3.93(2.13-6.15)	0.5%(-0.01-1.35)	0.35%(0.13-0.57)
Ethiopia	297(155-573)	1.48(0.79-2.8)	510(298-762)	1.29(0.75-1.93)	-0.18%(-0.48-0.51)	-0.61%(-0.74--0.49)
Fiji	9(6-12)	2.37(1.53-3.38)	17(11-24)	2.26(1.46-3.22)	0.64%(0.14-1.43)	-0.19%(-0.33--0.05)
Finland	571(414-730)	8.08(5.87-10.32)	771(513-1089)	6.39(4.23-9.04)	0.22%(-0.06-0.61)	-0.61%(-0.72--0.5)
France	6116(4311-7901)	7.95(5.67-10.33)	11166(7338-15732)	9.41(6.16-13.41)	0.59%(0.2-1.1)	0.82%(0.66-0.99)
Gabon	25(12-42)	4.24(2.19-7.05)	46(27-73)	4.32(2.51-6.64)	0.07%(-0.29-0.66)	-0.04%(-0.1-0.02)
Gambia	5(3-7)	1.41(0.91-2.06)	16(10-24)	1.73(1.11-2.53)	0.45%(-0.04-1.24)	0.55%(0.4-0.71)
Georgia	462(324-614)	7.13(4.97-9.52)	406(276-552)	7.15(4.84-9.78)	0.32%(0.01-0.7)	0.92%(0.39-1.46)
Germany	10000(7159-12934)	8.25(5.89-10.68)	15665(10467-22085)	9.07(6.06-12.93)	0.47%(0.11-0.95)	0.33%(0.08-0.58)
Ghana	123(82-173)	1.88(1.26-2.62)	327(210-469)	2.01(1.3-2.88)	0.27%(-0.13-0.81)	0.02%(-0.12-0.16)
Greece	1510(1069-1953)	9.94(7.01-12.81)	2173(1423-3071)	10.22(6.65-14.57)	0.45%(0.09-0.87)	0.02%(-0.08-0.11)
Greenland	7(5-9)	18.6(13.36-24.32)	12(8-16)	16.96(11.61-22.67)	0.78%(0.4-1.24)	-0.68%(-0.98--0.39)

location	Num_1990	ASR_1990	Num_2019	ASR_2019	Num_change	EAPC_CI
Grenada	2(2-3)	3.4(2.37-4.56)	4(3-5)	3.26(2.27-4.4)	0.32%(0.05-0.67)	0.06%(-0.08-0.21)
Guam	6(4-9)	8.35(5.79-11.18)	13(9-19)	7.04(4.79-9.69)	0.7%(0.3-1.24)	-0.57%(-0.79--0.35)
Guatemala	79(54-106)	2.12(1.44-2.86)	202(131-294)	1.8(1.17-2.61)	0.14%(-0.21-0.55)	-1.08%(-1.32--0.85)
Guinea	65(43-90)	1.95(1.3-2.68)	129(83-189)	2.32(1.5-3.39)	-0.03%(-0.35-0.38)	0.77%(0.72-0.81)
Guinea-Bissau	13(6-20)	3.09(1.58-4.76)	21(12-35)	2.94(1.66-4.68)	-0.12%(-0.42-0.33)	0.09%(-0.04-0.21)
Guyana	8(5-10)	1.95(1.33-2.66)	13(8-19)	2.02(1.25-2.93)	0.73%(0.21-1.44)	0.25%(0.15-0.34)
Haiti	105(53-182)	3.08(1.57-5.33)	183(101-315)	2.58(1.44-4.4)	-0.11%(-0.4-0.41)	-0.48%(-0.62--0.34)
Honduras	67(44-93)	3.17(2.07-4.39)	313(178-492)	5.15(2.92-8.04)	1.24%(0.47-2.29)	1.79%(1.66-1.92)
Hungary	1622(1163-2089)	11.25(8.08-14.51)	2174(1496-2962)	12.09(8.24-16.64)	0.44%(0.14-0.8)	0.11%(-0.17-0.4)
Iceland	25(17-32)	8.83(6.23-11.63)	48(33-65)	9.05(6.23-12.14)	0.45%(0.19-0.78)	0.08%(-0.14-0.29)
India	6898(4716-9363)	1.51(1.02-2.05)	20368(14190-27535)	1.77(1.23-2.38)	0.82%(0.33-1.29)	0.41%(0.31-0.51)
Indonesia	3825(2635-5095)	3.77(2.61-4.98)	10919(6938-15201)	5.01(3.21-6.99)	1.04%(0.48-1.59)	0.95%(0.9-0.99)

location	Num_1990	ASR_1990	Num_2019	ASR_2019	Num_change	EAPC_CI
Iran (Islamic Republic of)	675(459-919)	2.5(1.72-3.4)	2023(1445-2617)	2.78(1.99-3.59)	1.08%(0.64-1.59)	0.37%(0.19-0.55)
Iraq	277(181-390)	3.55(2.3-4.97)	957(630-1388)	4.17(2.74-5.92)	0.44%(-0.01-1.08)	0.75%(0.47-1.04)
Ireland	371(263-475)	9.01(6.4-11.55)	604(403-849)	8.11(5.4-11.42)	0.19%(-0.11-0.55)	-0.28%(-0.41--0.16)
Israel	256(182-333)	5.36(3.8-7.01)	634(414-902)	5.62(3.66-8.02)	0.32%(-0.03-0.76)	-0.11%(-0.23-0.01)
Italy	7852(5701-9991)	8.98(6.51-11.42)	10069(7038-13529)	7.57(5.24-10.21)	0.21%(-0.02-0.45)	-0.62%(-0.73--0.5)
Jamaica	62(43-83)	3.6(2.52-4.77)	132(85-191)	4.47(2.89-6.47)	0.79%(0.3-1.46)	0.79%(0.32-1.26)
Japan	13494(9904-16990)	7.9(5.81-9.96)	32090(22515-42646)	8.93(6.31-11.83)	1.34%(0.97-1.76)	0.68%(0.48-0.88)
Jordan	39(26-56)	2.85(1.94-3.99)	216(142-306)	3.26(2.17-4.61)	0.78%(0.27-1.49)	0.86%(0.64-1.08)
Kazakhstan	1316(915-1730)	9.76(6.8-12.8)	887(608-1204)	4.88(3.34-6.58)	-0.4%(-0.53--0.25)	-2.56%(-2.69--2.43)
Kenya	85(55-122)	1.05(0.68-1.51)	281(190-392)	1.3(0.88-1.81)	0.53%(0.22-0.96)	0.72%(0.61-0.84)
Kiribati	2(1-2)	4.31(2.92-5.93)	3(2-5)	4.28(2.76-6.33)	0.15%(-0.26-0.78)	-0.13%(-0.16--0.09)
Kuwait	17(11-22)	2.75(1.88-3.71)	54(36-75)	2.28(1.52-3.22)	0.28%(-0.05-0.68)	-0.44%(-0.74--0.14)

location	Num_1990	ASR_1990	Num_2019	ASR_2019	Num_change	EAPC_CI
Kyrgyzstan	195(136-258)	6.22(4.34-8.21)	136(92-187)	2.84(1.92-3.92)	-0.53%(-0.63--0.4)	-2.85%(-3.3--2.4)
Lao People's Democratic Republic	122(71-190)	5.61(3.36-8.5)	220(136-319)	5.01(3.1-7.19)	0.05%(-0.32-0.55)	-0.63%(-0.73--0.54)
Latvia	299(209-389)	8.31(5.81-10.8)	236(162-332)	6.37(4.37-8.96)	0.09%(-0.16-0.4)	-0.98%(-1.26--0.69)
Lebanon	109(69-159)	4.65(2.96-6.68)	320(214-461)	6.15(4.11-8.88)	0.85%(0.25-1.88)	1.63%(1.38-1.87)
Lesotho	28(18-43)	2.78(1.75-4.22)	53(30-81)	4(2.33-6.07)	0.62%(0.01-1.57)	1.48%(1.35-1.61)
Liberia	24(16-35)	2.18(1.43-3.14)	41(24-62)	2.08(1.24-3.16)	-0.31%(-0.55-0)	0.24%(-0.03-0.52)
Libya	84(55-119)	4.45(2.93-6.31)	212(138-306)	4.11(2.67-5.94)	0.59%(0.04-1.37)	-0.2%(-0.33--0.07)
Lithuania	361(256-478)	7.94(5.63-10.49)	320(219-443)	5.94(4.02-8.28)	0.17%(-0.1-0.51)	-0.98%(-1.18--0.78)
Luxembourg	55(39-72)	10.29(7.3-13.4)	78(52-107)	8.06(5.37-11.09)	-0.13%(-0.31-0.11)	-0.8%(-0.98--0.62)
Madagascar	83(53-117)	1.59(1.01-2.24)	165(104-247)	1.5(0.94-2.25)	-0.11%(-0.39-0.29)	-0.33%(-0.4--0.26)
Malawi	48(32-68)	1.28(0.83-1.78)	99(64-144)	1.4(0.91-1.99)	0.06%(-0.23-0.46)	0.27%(0.13-0.4)
Malaysia	341(235-463)	3.76(2.55-5.14)	1176(742-1703)	4.41(2.81-6.35)	0.94%(0.34-1.74)	0.44%(0.16-0.73)

location	Num_1990	ASR_1990	Num_2019	ASR_2019	Num_change	EAPC_CI
Maldives	3(1-4)	3.06(1.8-4.65)	6(4-9)	2.17(1.46-3.02)	0.07%(-0.32-0.75)	-1.68%(-1.86--1.5)
Mali	60(40-84)	1.45(0.98-2.01)	140(89-205)	1.66(1.07-2.42)	-0.08%(-0.37-0.33)	0.54%(0.49-0.59)
Malta	26(19-35)	6.1(4.35-8.02)	49(33-66)	5.47(3.69-7.42)	0.57%(0.22-0.96)	-0.23%(-0.3--0.16)
Marshall Islands	1(1-2)	5.96(3.12-9.77)	2(1-4)	6.58(3.55-10.48)	0.82%(0.26-1.6)	0.54%(0.43-0.65)
Mauritania	25(16-35)	2.44(1.57-3.48)	49(28-75)	2.43(1.43-3.62)	0.03%(-0.36-0.71)	0.26%(0.08-0.44)
Mauritius	24(17-32)	3.2(2.21-4.25)	47(31-67)	2.69(1.78-3.76)	0.69%(0.26-1.25)	-0.75%(-0.84--0.65)
Mexico	1320(942-1681)	3.13(2.23-3.98)	2525(1761-3333)	2.18(1.52-2.88)	0.31%(0.13-0.52)	-1.72%(-1.87--1.56)
Micronesia (Federated States of)	3(2-5)	6.11(3.72-9.5)	5(3-8)	6.9(3.72-11.06)	0.73%(0.08-1.61)	0.39%(0.36-0.42)
Monaco	7(5-10)	11.42(7.74-15.65)	16(11-22)	18.75(12.59-25.59)	0.84%(0.38-1.5)	2.1%(1.67-2.54)
Mongolia	92(62-127)	8.85(5.96-12.2)	151(98-218)	6.83(4.47-9.72)	0.04%(-0.27-0.5)	-1.6%(-1.85--1.34)
Montenegro	72(51-95)	11.17(8.01-14.72)	130(86-180)	13.17(8.67-18.21)	0.82%(0.4-1.35)	0.72%(0.57-0.87)
Morocco	477(307-668)	3.35(2.16-4.71)	1220(747-1808)	3.71(2.33-5.46)	0.8%(0.17-1.62)	0.24%(0.11-0.36)

location	Num_1990	ASR_1990	Num_2019	ASR_2019	Num_change	EAPC_CI
Mozambique	72(47-100)	1.25(0.81-1.72)	200(122-302)	1.87(1.16-2.81)	0.23%(-0.18-0.79)	1.75%(1.63-1.88)
Myanmar	1311(786-2173)	5.45(3.31-8.97)	2329(1472-3602)	4.97(3.19-7.61)	0.34%(-0.1-1.01)	-0.43%(-0.48--0.39)
Namibia	11(7-16)	1.47(0.97-2.1)	25(15-36)	1.77(1.12-2.53)	0.35%(-0.04-0.99)	0.65%(0.57-0.72)
Nauru	0(0-1)	8.76(5.26-13.44)	0(0-1)	8.38(4.75-12.57)	0.03%(-0.27-0.45)	-0.23%(-0.29--0.16)
Nepal	156(88-248)	1.64(0.91-2.66)	413(254-614)	1.85(1.14-2.74)	0.69%(0.07-1.5)	0.31%(0.13-0.49)
Netherlands	2313(1659-2968)	11.8(8.47-15.19)	3683(2485-5156)	11.12(7.43-15.71)	0.39%(0.06-0.78)	-0.02%(-0.22-0.18)
New Zealand	350(252-446)	8.92(6.44-11.39)	624(439-833)	8.07(5.66-10.8)	0.36%(0.08-0.67)	-0.36%(-0.45--0.28)
Nicaragua	27(18-37)	1.76(1.16-2.49)	85(57-119)	1.99(1.31-2.77)	0.91%(0.41-1.56)	0.33%(0.22-0.44)
Niger	54(31-85)	1.94(1.13-3.01)	154(84-243)	2.05(1.12-3.24)	-0.01%(-0.34-0.43)	0.44%(0.3-0.57)
Nigeria	623(383-940)	1.45(0.89-2.15)	1376(880-1970)	1.7(1.11-2.4)	-0.07%(-0.36-0.32)	0.86%(0.74-0.98)
Niue	0(0-0)	6.03(4.07-8.24)	0(0-0)	6.63(4.53-9.22)	0.55%(0.13-1.07)	0.33%(0.27-0.38)
North Macedonia	135(95-177)	6.78(4.79-8.89)	310(204-443)	9.34(6.15-13.35)	1.15%(0.58-1.93)	1.16%(0.95-1.37)

location	Num_1990	ASR_1990	Num_2019	ASR_2019	Num_change	EAPC_CI
Northern Mariana Islands	2(2-3)	12.31(8.63-16.92)	5(4-7)	9.84(6.94-13.03)	1.52%(0.91-2.36)	-0.91%(-1--0.83)
Norway	423(309-533)	6.63(4.82-8.35)	754(529-996)	8.03(5.63-10.63)	0.41%(0.19-0.68)	0.99%(0.67-1.32)
Oman	14(9-21)	2.1(1.3-3.12)	35(23-53)	2.21(1.46-3.15)	0.08%(-0.29-0.67)	0.44%(0.2-0.69)
Pakistan	1804(1203-2440)	3.14(2.1-4.24)	4259(2795-6129)	3.69(2.42-5.32)	0.19%(-0.19-0.71)	0.45%(0.23-0.67)
Palau	1(1-1)	9.23(6.02-13.13)	2(1-3)	9.68(6.66-13.46)	0.95%(0.34-1.73)	0.13%(0.09-0.17)
Palestine	39(24-58)	4.47(2.83-6.6)	120(82-163)	4.96(3.4-6.72)	0.28%(-0.13-0.89)	0.45%(0.23-0.66)
Panama	49(33-65)	3.27(2.2-4.33)	105(67-154)	2.56(1.62-3.73)	0.24%(-0.11-0.68)	-1.09%(-1.23--0.95)
Papua New Guinea	79(47-129)	4.14(2.44-6.81)	228(131-364)	4.76(2.81-7.5)	0.2%(-0.14-0.77)	0.53%(0.51-0.55)
Paraguay	54(37-74)	2.45(1.66-3.31)	197(124-290)	3.57(2.26-5.25)	1.12%(0.48-1.99)	1.2%(0.95-1.44)
Peru	411(281-565)	3.41(2.34-4.69)	777(485-1158)	2.43(1.51-3.62)	0.21%(-0.17-0.71)	-1.19%(-1.51--0.88)
Philippines	1566(1091-2027)	5.12(3.59-6.6)	3187(2120-4429)	3.98(2.67-5.49)	0.15%(-0.14-0.54)	-1.47%(-1.71--1.22)
Poland	4415(3165-5622)	10(7.15-12.72)	6570(4447-8871)	9.53(6.45-12.93)	0.48%(0.23-0.76)	-0.31%(-0.47--0.15)

location	Num_1990	ASR_1990	Num_2019	ASR_2019	Num_change	EAPC_CI
Portugal	617(431-819)	4.47(3.14-5.92)	1067(691-1551)	5.02(3.23-7.32)	0.64%(0.18-1.22)	0.49%(0.29-0.7)
Puerto Rico	140(98-184)	3.86(2.72-5.01)	206(133-293)	2.99(1.92-4.26)	0.51%(0.09-1)	-0.97%(-1.06--0.87)
Qatar	4(2-6)	3.81(2.43-5.38)	30(19-48)	3.96(2.61-5.78)	0.2%(-0.29-1.09)	0.41%(0.12-0.7)
Republic of Korea	1806(1291-2396)	5.72(4.09-7.55)	8771(6055-11979)	9.76(6.77-13.33)	3.03%(2.11-4.15)	1.3%(0.94-1.67)
Republic of Moldova	325(228-421)	6.94(4.9-9.01)	250(170-348)	4.29(2.93-5.94)	-0.07%(-0.26-0.16)	-1.03%(-1.46--0.6)
Romania	1774(1236-2344)	6.1(4.3-8.1)	2669(1820-3668)	7.8(5.29-10.84)	0.83%(0.39-1.34)	0.68%(0.53-0.84)
Russian Federation	14332(10422-18189)	7.62(5.52-9.69)	13687(9517-18089)	5.81(4.04-7.66)	-0.02%(-0.16-0.15)	-1.25%(-1.51--0.99)
Rwanda	60(38-88)	2.05(1.32-2.97)	111(64-183)	1.87(1.1-3.06)	0.04%(-0.35-0.83)	-0.78%(-0.98--0.57)
Saint Kitts and Nevis	1(1-1)	2.97(2.06-3.92)	2(1-3)	2.66(1.81-3.72)	0.16%(-0.14-0.56)	-0.53%(-0.72--0.34)
Saint Lucia	3(2-4)	3.11(2.19-4.14)	6(4-9)	2.84(1.92-3.98)	0.8%(0.39-1.29)	-0.48%(-0.7--0.26)
Saint Vincent and the Grenadines	2(1-2)	2.18(1.51-2.93)	3(2-4)	2.38(1.65-3.25)	1.02%(0.54-1.61)	0.19%(0.06-0.33)
Samoa	2(2-3)	2.59(1.7-3.68)	4(2-5)	2.59(1.66-3.69)	0.28%(-0.08-0.84)	-0.04%(-0.07--0.01)

location	Num_1990	ASR_1990	Num_2019	ASR_2019	Num_change	EAPC_CI
San Marino	3(2-4)	8.6(6.07-11.6)	5(4-8)	9.16(5.92-13.5)	0.37%(-0.03-0.91)	0.49%(0.39-0.58)
Sao Tome and Principe	2(1-3)	3.06(2.02-4.25)	4(3-6)	4.17(2.73-5.96)	0.28%(-0.08-0.83)	1.07%(1.01-1.13)
Saudi Arabia	100(63-146)	1.74(1.11-2.54)	382(252-554)	2.02(1.31-2.9)	0.71%(0.13-1.67)	0.49%(0.37-0.61)
Senegal	79(51-115)	2.45(1.58-3.55)	200(125-294)	2.71(1.72-3.98)	0.27%(-0.13-0.87)	0.66%(0.43-0.89)
Serbia	1095(766-1442)	8.98(6.26-11.8)	1771(1174-2490)	11.55(7.62-16.25)	0.74%(0.27-1.39)	1.09%(0.92-1.26)
Seychelles	2(2-3)	4.15(2.85-5.55)	4(3-6)	3.92(2.67-5.2)	0.32%(0.02-0.71)	-0.47%(-0.61--0.34)
Sierra Leone	42(27-61)	2.21(1.43-3.2)	83(50-125)	2.36(1.44-3.54)	-0.13%(-0.4-0.24)	0.54%(0.35-0.74)
Singapore	217(156-281)	10(7.21-12.96)	610(414-850)	7.89(5.36-10.98)	0.51%(0.17-0.97)	-0.82%(-0.92--0.73)
Slovakia	663(473-859)	11.1(7.88-14.44)	788(509-1132)	8.51(5.48-12.22)	0.15%(-0.15-0.54)	-0.8%(-1.11--0.48)
Slovenia	204(131-296)	8.3(5.3-11.98)	332(217-468)	8.28(5.43-11.73)	0.55%(0.02-1.27)	-0.01%(-0.21-0.19)
Solomon Islands	10(4-17)	6.53(3.23-11.5)	24(11-41)	7.24(3.59-12.31)	0.29%(-0.11-0.86)	0.33%(0.26-0.4)
Somalia	39(22-66)	1.55(0.86-2.52)	88(42-160)	1.3(0.63-2.36)	-0.22%(-0.53-0.23)	-0.54%(-0.6--0.48)

location	Num_1990	ASR_1990	Num_2019	ASR_2019	Num_change	EAPC_CI
South Africa	1063(689-1593)	4.94(3.18-7.46)	1924(1343-2570)	4.25(2.98-5.65)	0.2%(-0.03-0.44)	-0.75%(-1.05--0.46)
South Sudan	53(30-88)	2.23(1.28-3.68)	73(41-115)	1.97(1.11-3.03)	-0.13%(-0.42-0.34)	-0.44%(-0.5--0.39)
Spain	4402(3198-5739)	8.26(6.01-10.81)	7497(4926-10691)	8.66(5.63-12.5)	0.44%(0.08-0.88)	0.26%(0.14-0.37)
Sri Lanka	204(138-276)	1.82(1.23-2.43)	594(358-902)	2.28(1.37-3.45)	1.3%(0.56-2.38)	1.42%(1.21-1.62)
Sudan	168(80-333)	1.79(0.86-3.52)	364(211-611)	1.92(1.12-3.28)	0.07%(-0.32-0.81)	0.28%(0.23-0.32)
Suriname	8(5-11)	3.02(2.06-4.1)	21(14-29)	3.44(2.27-4.84)	0.78%(0.34-1.36)	0.48%(0.25-0.71)
Sweden	671(479-878)	4.8(3.41-6.24)	978(686-1317)	4.83(3.37-6.46)	0.22%(-0.02-0.49)	0.05%(-0.1-0.2)
Switzerland	929(670-1191)	9.47(6.84-12.14)	1071(704-1529)	6.57(4.33-9.36)	-0.1%(-0.32-0.2)	-0.96%(-1.13--0.79)
Syrian Arab Republic	131(84-187)	2.36(1.51-3.39)	327(201-485)	2.57(1.59-3.72)	1.23%(0.46-2.46)	0.3%(0.12-0.48)
Taiwan (Province of China)	940(661-1254)	5.71(4.05-7.58)	2968(1984-4191)	7.51(5.05-10.57)	1.73%(1.06-2.58)	0.8%(0.44-1.16)
Tajikistan	136(92-179)	4.57(3.12-5.99)	143(93-207)	2.76(1.83-3.92)	-0.4%(-0.56--0.19)	-1.13%(-1.59--0.66)
Thailand	2407(1671-3191)	6.46(4.54-8.54)	5127(3159-7512)	5.03(3.14-7.33)	0.73%(0.2-1.46)	-1.32%(-1.49--1.15)

location	Num_1990	ASR_1990	Num_2019	ASR_2019	Num_change	EAPC_CI
Timor-Leste	10(7-16)	3.59(2.34-5.36)	35(22-52)	4.27(2.68-6.17)	1%(0.3-2.05)	0.74%(0.51-0.96)
Togo	29(19-41)	2.34(1.52-3.38)	92(59-138)	2.54(1.63-3.77)	0.48%(0.01-1.14)	0.35%(0.3-0.4)
Tokelau	0(0-0)	4.79(3.05-6.83)	0(0-0)	5.37(3.5-7.91)	0.31%(-0.08-0.89)	0.44%(0.41-0.48)
Tonga	3(2-5)	5.91(3.96-8.5)	5(3-7)	6.11(4.03-8.49)	0.39%(0-0.95)	0.06%(-0.1-0.21)
Trinidad and Tobago	23(16-30)	2.71(1.88-3.57)	46(28-68)	2.44(1.52-3.62)	0.71%(0.18-1.4)	-0.43%(-0.53--0.33)
Tunisia	218(143-305)	4.25(2.79-5.93)	557(330-850)	4.33(2.59-6.57)	0.87%(0.21-1.92)	-0.18%(-0.28--0.07)
Turkey	3194(2098-4493)	8.52(5.68-11.9)	6702(4548-9478)	7.46(5.05-10.51)	0.54%(0.05-1.25)	0.19%(-0.53-0.92)
Turkmenistan	85(59-112)	4.01(2.79-5.3)	102(65-147)	2.39(1.54-3.42)	-0.13%(-0.38-0.2)	-2.11%(-2.68--1.55)
Tuvalu	0(0-1)	5.35(3.44-8.35)	1(0-1)	5.71(3.58-8.54)	0.23%(-0.13-0.74)	0.17%(0.14-0.2)
Uganda	89(57-125)	1.38(0.89-1.92)	239(152-336)	1.69(1.08-2.39)	0.13%(-0.19-0.61)	0.59%(0.5-0.67)
Ukraine	7364(5256-9409)	10.17(7.25-12.98)	5007(3354-6839)	6.83(4.58-9.31)	-0.19%(-0.38-0.03)	-2.28%(-2.7--1.85)
United Arab Emirates	15(9-21)	4.09(2.57-5.92)	131(80-199)	3.96(2.41-6.22)	0.82%(0.1-1.94)	-0.15%(-0.52-0.22)

location	Num_1990	ASR_1990	Num_2019	ASR_2019	Num_change	EAPC_CI
United Kingdom	10365(7489-13047)	11.51(8.35-14.47)	12524(8851-16513)	9.96(7.04-13.24)	0.03%(-0.13-0.22)	-0.43%(-0.52--0.34)
United Republic of Tanzania	209(124-319)	1.9(1.15-2.88)	497(298-818)	2.05(1.23-3.33)	0.09%(-0.24-0.5)	0.21%(0.15-0.26)
United States of America	43934(32387-54837)	14.19(10.45-17.73)	61843(43851-80365)	11.09(7.86-14.45)	0.09%(-0.05-0.26)	-1.16%(-1.33--0.99)
United States Virgin Islands	4(2-5)	4.07(2.82-5.62)	10(7-14)	5.49(3.73-7.63)	1.95%(1.21-2.97)	1.44%(1.2-1.67)
Uruguay	381(271-492)	9.98(7.06-12.89)	398(255-568)	7.89(5.05-11.4)	-0.05%(-0.3-0.25)	-0.79%(-0.9--0.67)
Uzbekistan	516(358-691)	4.27(2.95-5.67)	673(444-943)	2.94(1.96-4.05)	-0.19%(-0.39-0.06)	-1.74%(-2--1.47)
Vanuatu	3(2-5)	4.6(2.57-7.67)	9(6-15)	5.43(3.24-8.71)	0.61%(0.08-1.74)	0.47%(0.34-0.6)
Venezuela (Bolivarian Republic of)	423(299-555)	4.28(2.98-5.59)	1340(870-1991)	4.55(2.98-6.75)	1.12%(0.53-1.87)	0.31%(0.1-0.53)
Viet Nam	1994(1318-2777)	4.83(3.21-6.69)	5927(3923-8368)	6.08(4.03-8.53)	1.1%(0.45-2.02)	0.83%(0.81-0.85)
Yemen	110(59-191)	2.18(1.2-3.73)	308(181-505)	2.29(1.35-3.77)	0.22%(-0.23-0.96)	0.35%(0.29-0.41)
Zambia	65(42-93)	2.28(1.48-3.23)	165(105-247)	2.45(1.56-3.61)	0.11%(-0.24-0.58)	0.04%(-0.04-0.11)
Zimbabwe	127(86-174)	3.05(2.06-4.13)	237(157-339)	3.27(2.18-4.68)	0.28%(-0.08-0.76)	0.29%(0.15-0.43)
